# A LlMYB305-LlC3H18-LlWRKY33 module regulates thermotolerance in lily

**DOI:** 10.1186/s43897-023-00064-1

**Published:** 2023-08-17

**Authors:** Ze Wu, Jiahui Liang, Ting Li, Dehua Zhang, Nianjun Teng

**Affiliations:** 1 Key Laboratory of Landscaping, Ministry of Agriculture and Rural Affairs, Key Laboratory of Biology of Ornamental Plants in East China, National Forestry and Grassland Administration, College of Horticulture, Nanjing Agricultural University, Nanjing, 210095 China; 2 Jiangsu Graduate Workstation of Nanjing Agricultural University and Nanjing Oriole Island Modern Agricultural Development Co., Ltd, Nanjing, 210043 China; 3 College of Agriculture, Nanjing Agricultural University, Nanjing, 210095 China; 4 Institute of Grassland, Flowers and Ecology, Beijing Academy of Agriculture and Forestry Sciences, Beijing, 100097 China

**Keywords:** CCCH-zinc finger protein, *Lilium longiflorum*, LlC3H18, LlMYB305, LlWRKY33, Thermotolerance

## Abstract

The CCCH proteins play important roles in plant growth and development, hormone response, pathogen defense and abiotic stress tolerance. However, the knowledge of their roles in thermotolerance are scarce. Here, we identified a heat-inducible *CCCH* gene *LlC3H18* from lily. LlC3H18 was localized in the cytoplasm and nucleus under normal conditions, while it translocated in the cytoplasmic foci and co-located with the markers of two messenger ribonucleoprotein (mRNP) granules, processing bodies (PBs) and stress granules (SGs) under heat stress conditions, and it also exhibited RNA-binding ability. In addition, LlC3H18 exhibited transactivation activity in both yeast and plant cells. In lily and Arabidopsis, overexpression of *LlC3H18* damaged their thermotolerances, and silencing of *LlC3H18* in lily also impaired its thermotolerance. Similarly, Arabidopsis *atc3h18* mutant also showed decreased thermotolerance. These results indicated that the appropriate expression of *C3H18* was crucial for establishing thermotolerance. Further analysis found that LlC3H18 directly bound to the promoter of *LlWRKY33* and activated its expression. Besides, it was found that LlMYB305 acted as an upstream factor of *LlC3H18* and activated its expression. In conclusion, we demonstrated that there may be a LlMYB305-LlC3H18-LlWRKY33 regulatory module in lily that is involved in the establishment of thermotolerance and finely regulates heat stress response.

**Graphical Abstract:**

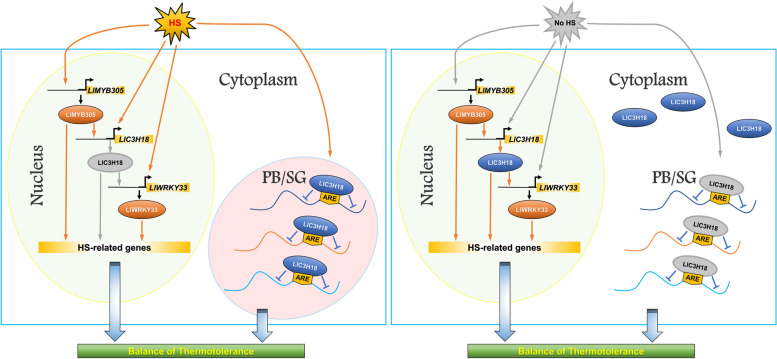

**Supplementary Information:**

The online version contains supplementary material available at 10.1186/s43897-023-00064-1.

## Core

Lily heat-inducible *CCCH* gene *LlC3H18* is directly activated by LlMYB305, and its protein is partially localized in the nucleus to act as a transcription activator of *LlWRKY33*, thus forming a LlMYB305-LlC3H18-LlWRKY33 regulatory module. LlC3H18 can also locate in the cytoplasm foci under high temperature conditions, and play a role of RNA-binding protein to form mRNP granules for finely regulating heat stress response.

## Gene and accession numbers

Sequence data from this article can be found in the database of the National Center for Biotechnology Information (NCBI) under the accession numbers: LlC3H18 (OR094243), LlMYB305 (MW383251), LlWRKY33 (OR094247).

## Introduction

With the development of industry, a large amount of fossil energy is used, the emission of carbon dioxide increases year by year, the trend of global warming is inevitable, and more and more abnormally high temperature weather occurs frequently (Grover et al. 2013; Wahid et al. 2007). As sessile organisms, plants are difficult to escape the adverse effects of environmental changes and are extremely sensitive to temperature changes, especially for some horticultural crops and food crops; high temperature often leads to reduced yield and quality (Teixeira et al. 2013). Many studies have proved that transcription factors (TFs) play an important role in the regulation of plant thermotolerance (Ohama et al. 2017). It may be feasible to improve thermotolerance in crops by screening heat-resistant regulators and genetic engineering methods.

The CCCH proteins are a class of proteins with zinc finger (ZNF) domains, many of which can function as TFs to regulate the expression of target genes (Pomeranz et al. 2011; Wang et al. 2008). The CCCH proteins contain 1–6 CCCH-type ZNF motifs which is consist of three Cys residues and one His residue (C-X_5-14_-C-X_4-5_-C-X_3_-H)(Wang et al. 2008). In Arabidopsis, there are 68 CCCH members and they are classified into 11 subfamilies based on the spacing between Cys and His in the ZNF motifs as well as the number of ZNF motifs (Wang et al. 2008). In addition, CCCH proteins are divided into tandem CCCH-type zinc finger (TZF) and non-TZF proteins: TZF proteins contain two tandem CCCH-type ZNF motifs whereas non-TZF proteins have fewer or greater than two CCCH-type ZNF motifs (Bogamuwa and Jang 2014; Seok et al. 2018). Except the ZNF domain, some members of CCCH family also had a LOTUS/OST-HTH (Limkain, Oskar, and TUdor-containing proteins 5 and 7) domain and an RRM (RNA-recognition motif) domain, both are putative RNA-binding domains, so they are always putative RNA-binding proteins for post-transcriptional regulation (Pomeranz et al. 2010b; Xu et al. 2022). For AtTZF1, the TZF motif is important for RNA binding in a zinc-dependent fashion (Pomeranz et al. 2010b; Qu et al. 2014). Moreover, AtCPSF30/AtC3H11 and AtSmicl bind to RNA and have nuclease activity (Addepalli and Hunt 2008). However, it was also reported that plant CCCH proteins function in transcriptional regulation. AtC3H14 and AtC3H15 regulate transcription through DNA-binding and they exhibit transactivation activity in yeast (Chai et al. 2015). In plants, CCCH proteins are a kind of regulators playing significant roles in plant growth, development, hormone response, defense pathogens, and resist to abiotic stresses (Bogamuwa and Jang 2014; Han et al. 2021). AtTZF2/AtOZF1, AtTZF3/AtOZF2, and cotton GhZFP1 are all associated with jasmonic acid-induced leaf senescence (Guo et al. 2009; Lee et al. 2012). AtTZF4, AtTZF5, and AtTZF6 positively regulate abscisic acid (ABA) response and play roles in seed germination and embryo formation, and AtC3H17 has pleiotropic effects on vegetative development, flowering, and seed development in Arabidopsis (Bogamuwa and Jang 2013; Seok et al. 2016). AtC3H14 and AtC3H15 are involved in the regulation of cell elongation, secondary wall thickening, male fertility, anther development, and acquisition of immunity against pathogens (Chai et al. 2015; Wang et al. 2022a, 2020). Poplar PdC3H17 and PdC3H18 positively regulate secondary wall formation in poplar (Chai et al. 2014). AtC3H11 is a subunit of polyadenylation factor and is required for *Pseudomonas* resistance (Bruggeman et al. 2014). Pepper CaC3H14 positively regulates the response of inoculation by *Ralstonia solanacearum* (Qiu et al. 2018). OsLIC promotes downstream *OsWRKY30* for rice resistance to bacterial blight and leaf streak (Wang et al. 2022b). In addition, a number of CCCH proteins, such as AtTZF1, GhZFP1, GhTZF1, OsTZF1, AtSZF1/2, OsC3H47, PvC3H72, and OsDOS were found as important regulators for plant responses to salt, drought, cold, and oxidative stresses (Guo et al. 2009; Jan et al. 2013; Kong et al. 2006; Lin et al. 2011; Sun et al. 2007; Wang et al. 2015; Xie et al. 2019; Zhou et al. 2014). AtSZF1/AtTZF11 and AtSZF2/AtTZF10 negatively regulate salt stress response (Sun et al. 2007), whereas AtC3H17 functions as a positive regulator in salt stress response (Seok et al. 2018). Overexpression of *AtTZF2*/*AtOZF1* or *AtTZF3*/*AtOZF2* has shown to confer ABA hypersensitivity and drought tolerance (Lee et al. 2012). In rice, the expression of *OsTZF1* is up-regulated by drought, salt stress, and hydrogen peroxide, which overexpression improves tolerance to salt and drought stresses and vice versa for knockdown plants (Jan et al. 2013). Overexpression of cotton *GhZFP1* enhances tolerance to drought and delays drought-induced senescence (Guo et al. 2009). The *atc3h11* mutant alters the poly(A) site choice and mRNA profile, and enhances the tolerance to oxidative stress (Hunt et al. 2008). Functional studies have revealed that some CCCH proteins are engaged in the regulation of abiotic stress responses. However, there has been no report on CCCH proteins’ involvement in plant thermotolerance and signal transduction to date.

Here, we reported the isolation and functional characterization of lily *LlC3H18*, which was induced under heat stress (HS) conditions. LlC3H18 was co-localized with processing body (PB) and stress granule (SG) markers under HS conditions, and it also showed transactivation activity in yeast and plant cells. LlC3H18 could activate the expression of *LlWRKY33* by binding its promoter. Further analysis showed that the appropriate expression of *LlC3H18* played a required role in thermotolerance, and it might function as a target of LlMYB305.

## Results

### Lily *LlC3H18* encodes a non-TZF CCCH protein that is activated by high temperature

**Fig. 1:**
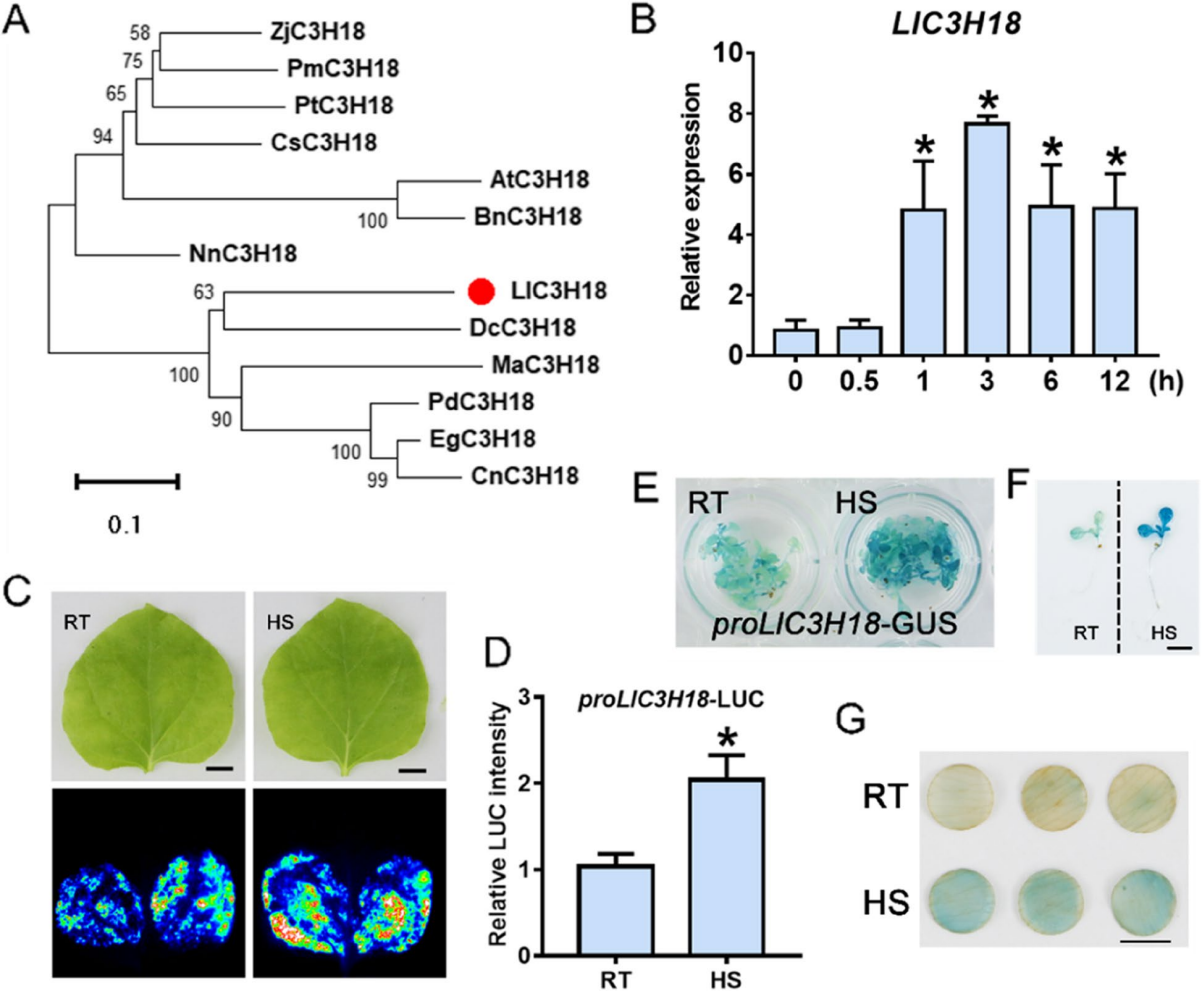
LlC3H18 is a heat-inducible CCCH-type protein. **A** Phylogenetic tree analysis of LlC3H18 and its homologs from other plant species. The evolutionary tree was assembled in MEGA 7.0 via the neighbor-joining method (bootstrap replicates, *n* = 1,000). PdC3H18 (*Phoenix dactylifera*, XP_008800632.1); EgC3H18 (*Elaeis guineensis*, XP_010907182.1); DcC3H18 (*Dioscorea cayenensis*, XP_039146376.1); CnC3H18 (*Cocos nucifera*, KAG1342178.1); NnC3H18 (*Nelumbo nucifera*, XP_010270169.1); MaC3H18 (*Musa acuminata*, XP_009394377.1); ZjC3H18 (*Ziziphus jujuba*, XP_048327362.1); CsC3H18 (*Camellia sinensis*, XP_028094358.1); AtC3H18 (*Arabidopsis thaliana*, AT2G05160); PtC3H18 (*Populus trichocarpa*, XP_024441083.1); PmC3H18 (*Prunus mume*, XP_008229903.1); BnC3H18 (Brassica napus, XP_048619457.1). **B** The expression of *LlC3H18* in lily leaves under heat stress conditions for different time durations. HS, heat stress, 37°C. Bars indicate the mean ± SD from three replicates (Student’s *t*-test, * *P* < 0.05, all treatments compared with 0 h). **C** The LUC reporter assay of *LlC3H18* promoter activity in tobacco leaves at room temperature (RT, 22°C) and under HS (37°C, 3 h). One representative image based on three independent experiments. Scale bar = 1 cm. **D** Quantification of LUC intensity in panel C. All values shown are the mean ± SD of three replicates (Student’s *t*-test, * *P* < 0.05). **E** The activity of *LlC3H18* promoter in *proLlC3H18*-GUS transgenic Arabidopsis at RT (22°C) and under HS (37°C, 3 h). One representative image based on three replicates. **F** The single plant of proLlC3H18-GUS transgenic Arabidopsis in (E). Scale bar = 1 cm. **G** *LlC3H18* promoter activity in *proLlC3H18*-GUS that was transiently expressed lily petal discs at RT (22°C) and under HS (37°C, 3 h). One representative image based on three independent experiments. Scale bar = 1 cm

By analyzing the transcriptome data of HS-treated lily leaves, we obtained a CCCH-type gene, *LlC3H18*, which was differentially expressed under normal and high temperature conditions (Fig. S1). Based on the transcriptome data, the ORF of *LlC3H18* was cloned from lily ‘White heaven’, and it was 1728 bp and was speculated to encode a non-TZF protein containing 575 amino acids. Through phylogenetic tree analysis with the CCCH proteins of Arabidopsis, the results showed that LlC3H18 is most closely related to AtC3H18 (Fig. S2), so it was named LlC3H18. The phylogenetic tree analysis was performed with C3H18 homologies from other plant species, the results showed that LlC3H18 clustered with monocots’ C3H18 and was most closely related to EgC3H18 of oil palm (*Elaeis guineensis*) (Fig. 1A). Alignment of protein domains found that LlC3H18 contained a conserved C-X_7_-C-X_5_-C-X_3_-H type CCCH domain and two putative RBDs (RNA-binding domains), LOTUS (Limkain, Oskar, and TUdor-containing proteins 5 and 7) and RRM (RNA-recognition motif) (Fig. S3). After HS treatment, the expression of *LlC3H18* was continuously activated by high temperature in lily leaves (Fig. 1B). The *LlC3H18* promoter was isolated from lily, and its activity was analyzed by transient transformation in tobacco leaves (Fig. 1C). After HS, the expression of the *LUC* reporter driven by the *LlC3H18* promoter increased significantly (Fig. 1C, D). In addition, a promoter-driven *proLlC3H18*-GUS reporter vector was also constructed, and the GUS transgenic Arabidopsis line was obtained; it was observed that high temperature could activate the activity of *LlC3H18* promoter (Fig. 1E, F). According to the transient GUS reporter assay in lily petal discs, it was observed that the *proLlC3H18*-GUS activity could be evidently activated by HS (Fig. 1G). Therefore, these data indicated that LlC3H18 is a heat-inducible CCCH-type protein in lily.

### LlC3H18 localizes in cytoplasmic foci in response to heat stress

**Fig. 2:**
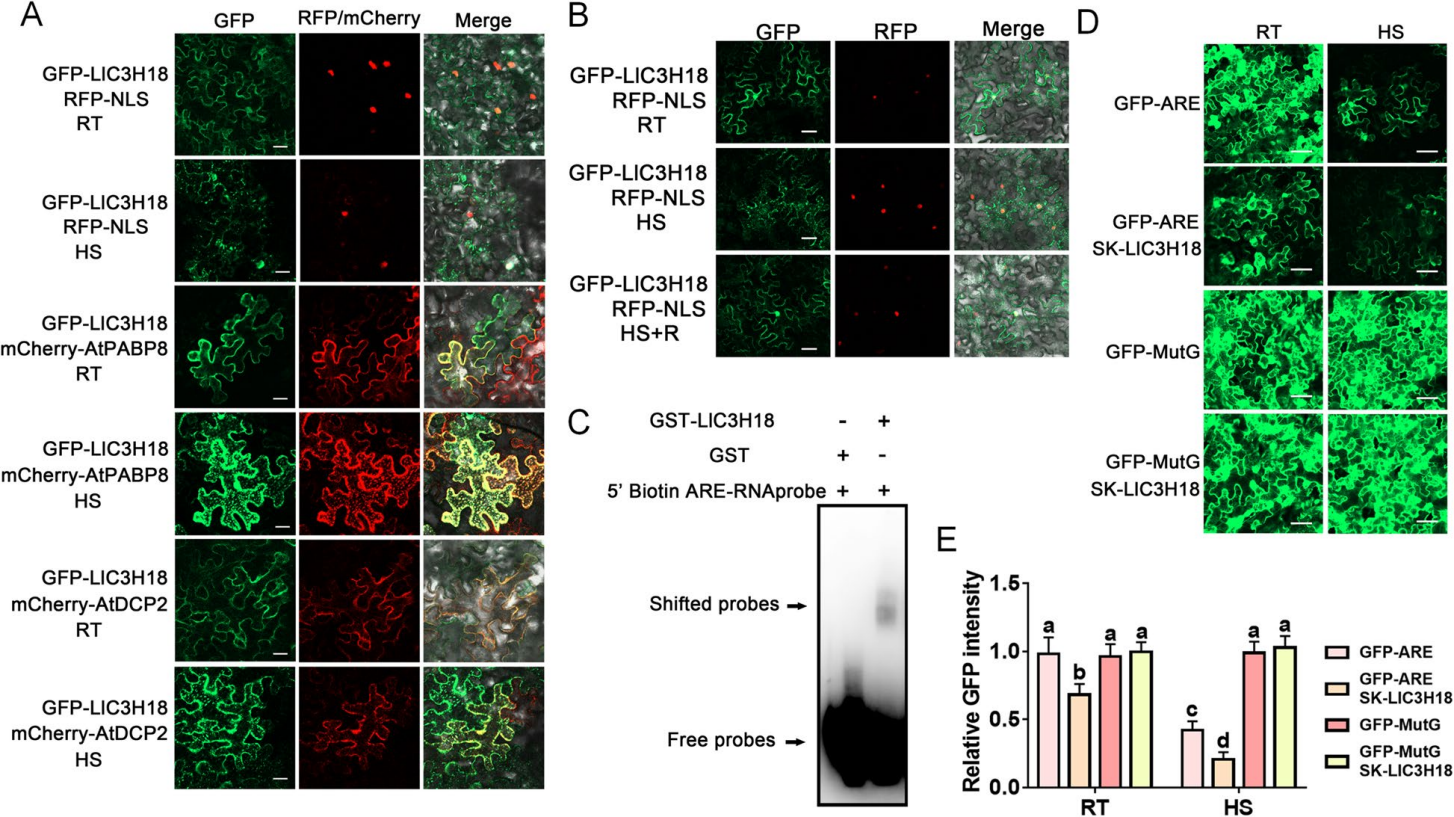
Subcellular localization assay of LlC3H18. **A** Detection of fluorescence signals in tobacco leaf cells co-transfected with GFP-LlC3H18, the nuclear marker RFP-NLS, the PB marker RFP-AtDCP2, the SG marker RFP-AtPABP8 at room temperature (RT, 22°C) and under HS (37°C, 3 h). Scale bar = 50 µm. **B** Detection of fluorescence signals in tobacco leaf cells co-transfected with GFP-LlC3H18, and the nuclear marker RFP-NLS at room temperature (RT, 22°C), under HS (37°C, 3 h), and after recovery 1 h from HS (HS + R, 22°C). Scale bar = 50 µm. **C** RNA-EMSA assay of GST- LlC3H18 protein and ARE sequence. One representative image based on three independent experiments. **D** GFP-ARE or GFP-MutG co-transformed with LlC3H18. GFP-MuG (replace the A residue in ARE with G) as a negative control. RT, room temperature, 22°C; HS, heat stress, 37°C, 3 h. Scale bar = 50 µm. **E** The GFP intensity in (D) is measured. Data are presented as the mean ± SD of three replicates, with different letters indicating statistically significant difference (Student–Newman–Keuls test, *P* < 0.05)

With transiently expression of GFP-LlC3H18 in tobacco leaves, in addition to the part GFP signal in the nucleus, which was consistent with the distribution of the RFP fluorescence of the nucleus marker RFP-NLS, but the fluorescence signal of GFP-LlC3H18 was also distributed throughout the cytoplasm (Fig. 2A). After HS, excitingly, it was observed high temperature changed GFP-LlC3H18 from being mainly dispersed in the cytoplasm to being aggregated into cytoplasmic foci, which was consistent with the distribution of the mCherry fluorescence of the PB marker mCherry-DCP2 and the SG maker mCherry-PABP8 (Fig. 2A) (Decker and Parker 2012; Xu et al. 2022). Due to both PB and SG are the mRNP granules, so these results indicated that LlC3H18 exhibited variable subcellular localization characteristics and may play roles in mRNA regulation with RNA-binding ability. Simultaneously, LlC3H18 also localized in nucleus under normal conditions or the recovery period after HS (Fig. 2B), which implicated a nucleus–cytoplasm shuttling process for LlC3H18-mediated signaling pathways and LlC3H18 might also function as a TF with DNA-binding ability. More CCCH proteins are known to regulate target genes by modulating the stability of mRNAs containing AU-rich element (ARE) in the 3’-UTRs (Pomeranz et al. 2010b; Qu et al. 2014; Xu et al. 2023). Then, we performed in vitro RNA-binding assays using GST-LlC3H18 proteins. The result of EMSA showed that LlC3H18 could bind to the labeled RNA fragment of ARE (Fig. 2C). To test whether LlC3H18 has the function to modulate the RNA stability in vivo, we created an ARE transcript mimic by fusing ARE to the GFP coding sequence (referred to as GFP-ARE). The A residues in ARE of GFP-ARE were replaced with G to generate a negative control (referred to as GFP-MutG) (Brewer et al. 2004). We next tested the effect of ARE on GFP mRNA translation. Co-expressing LlC3H18 and GFP-ARE in tobacco leaves generate much lower GFP fluorescence than expressing GFP-ARE alone (Fig. 2D, E). However, co-expressing mCherry-LlC3H18 and GFP-MutG had no effect on the accumulation of GFP fluorescence compared to those expressing GFP-MutG alone (Fig. 2D, E). These results indicated that LlC3H18 was able to bind RNA.

### LlC3H18 exhibits transactivation activity in yeast and plant cells

**Fig. 3:**
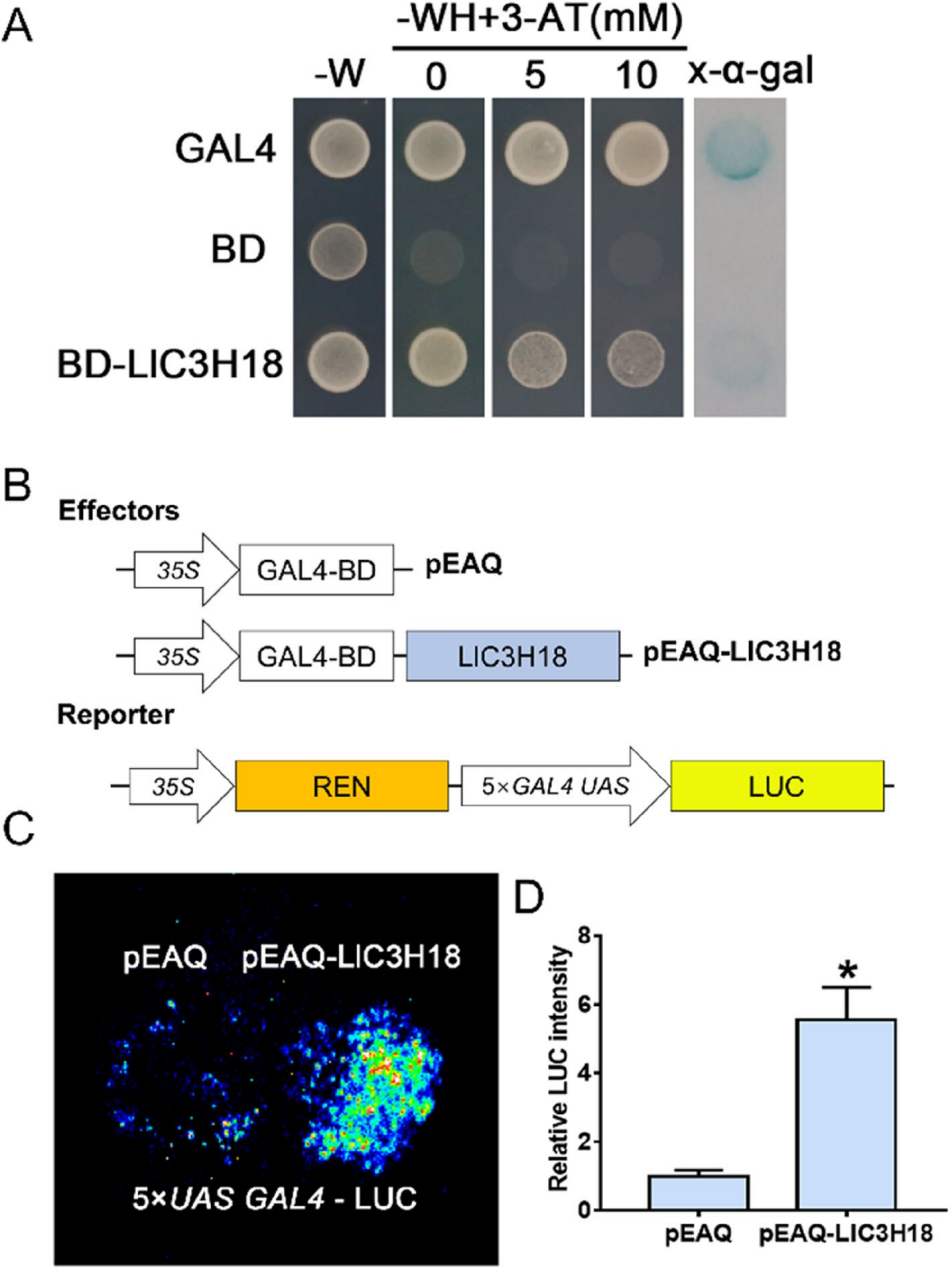
Transactivation assay of LlC3H18. **A **Transactivation activity assay in the yeast AH109 strain. The transformants were screened on SD-W medium (lacking Trp) while the growth of transformants was detected on SD-WH medium (lacking Trp/His) containing 3-amino-1,2,4-triazole (3-AT). The color reaction associated with x-α-gal degradation was used as a readout for β-galactosidase activity in the transformants. Representative image based on three replicates. **B** The constructs for the LUC reporter assay. **C** Detection of the LUC signal in infiltrated tobacco leaves. The image is representative of three independent experiments. Scale bar = 1 cm. **D** Measurement of LUC intensity in the reporter assay (Student’s *t*-test, * *P* < 0.05)

The BD vector was constructed to test whether LlC3H18 has transactivation activity in yeast. It was observed that yeast cells containing LlC3H18 grew well on the –WH plates and catalyzed the degradation of *β*-galactosidase (Fig. 3A), indicating that LlC3H18 showed transactivation activity in yeast. At the same time, pEAQ-BD and GAL4-LUC reporter vectors were constructed, and the transcriptional activity of LlC3H18 was transiently detected in tobacco leaves (Fig. 3B, C). The BD-LlC3H18 expression in tobacco leaves exhibited a stronger LUC signal compared with the expression of GAL4-BD only (Fig. 3D). This result suggested that LlC3H18 also had transactivation activity in tobacco cells.

### Overexpression of *LlC3H18* causes growth defects in transgenic plants

**Fig. 4:**
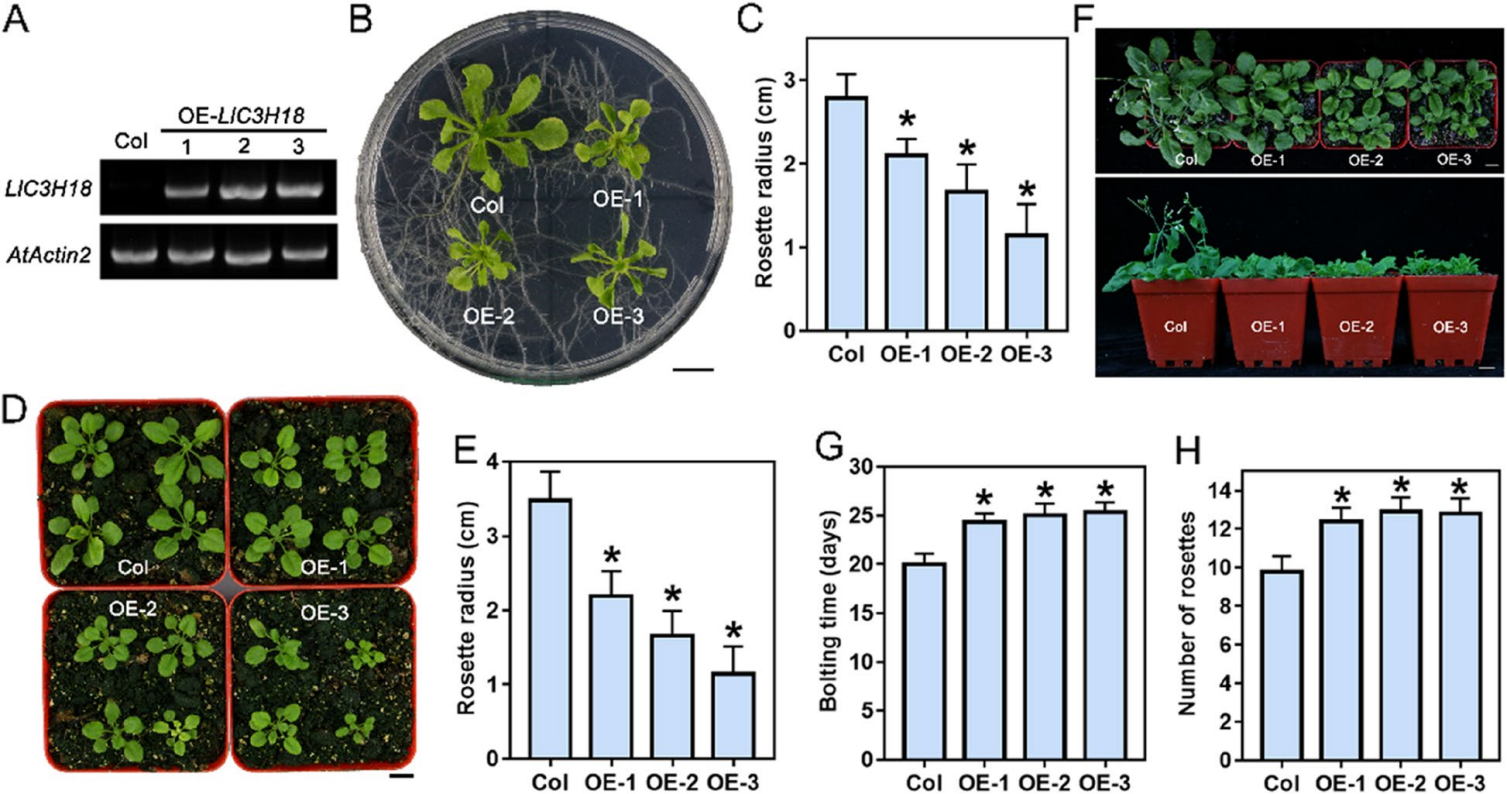
Overexpression of *LlC3H18* causes growth defectives. **A** Detection of *LlC3H18*-overexpression lines by RT-PCR. The 5-day-old seedlings were used to detect the expression of *LlC3H18* in transgenic Arabidopsis lines. PCR of the endogenous control and test gene was performed with 28 and 30 cycles, respectively. *AtActin2* was used as an endogenous control. **B** Seedlings of wild-type and transgenic lines grown on MS medium for 3 weeks. Scale bar = 1 cm. **C** Rosette radii of the plants which grown on MS medium for 3 weeks were counted. Bars are means ± SD of the tested plants (*n* = 9). **D** The 10-days-old seedlings were transferred from agar plates to soil for two weeks. The representative picture based on three replicates. Scale bar = 1 cm. **E** Rosette radii of the plants which grown on the soil for two weeks were counted. Bars are means ± SD of the tested plants (*n* = 9). **F** The 10-days-old seedlings were transferred from agar plates to soil for three weeks. Scale bar = 1 cm. The representative picture based on three replicates. **G** Bolting time for wild-type and transgenic lines. Bars are means ± SD of three independent experiments (n = 9, Student’s *t*-test, *P < 0.05). **H** The number of rosettes of the bolting transgenic and wild-type plants. Bars are means ± SD of three independent experiments (*n* = 9, Student’s *t*-test, *P < 0.05)

For exploring the function of LlC3H18 in vivo, *LlC3H18* driven by 35S promoter was stably transformed into Arabidopsis plants, and three overexpression lines were obtained by RT-PCR and selected for later functional analysis (Fig. 4A). The 5-day-old seedlings were transferred to MS medium to observe their growth (Fig. 4B). Obviously, compared to the wild-type plants, the overexpression plants grew more slowly, had smaller rosette leaves (Fig. 4B, C). The 7-day-old seedlings were transferred to the culture substrate for observing their growth (Fig. 4D). Likewise, the overexpression plants still exhibited growth defects, with smaller rosette leaves (Fig. 4D, E). In addition, we also observed that the overexpression plants showed a pronounced phenotype of delayed flowering, which required longer growth time and more rosette leaves to flowering (Fig. 4F-H). Therefore, these results suggested that constitutive overexpression of *LlC3H18* might impair normal growth and development, resulting in growth defects.

### Overexpression of *LlC3H18* damages thermotolerance of transgenic plants

**Fig. 5:**
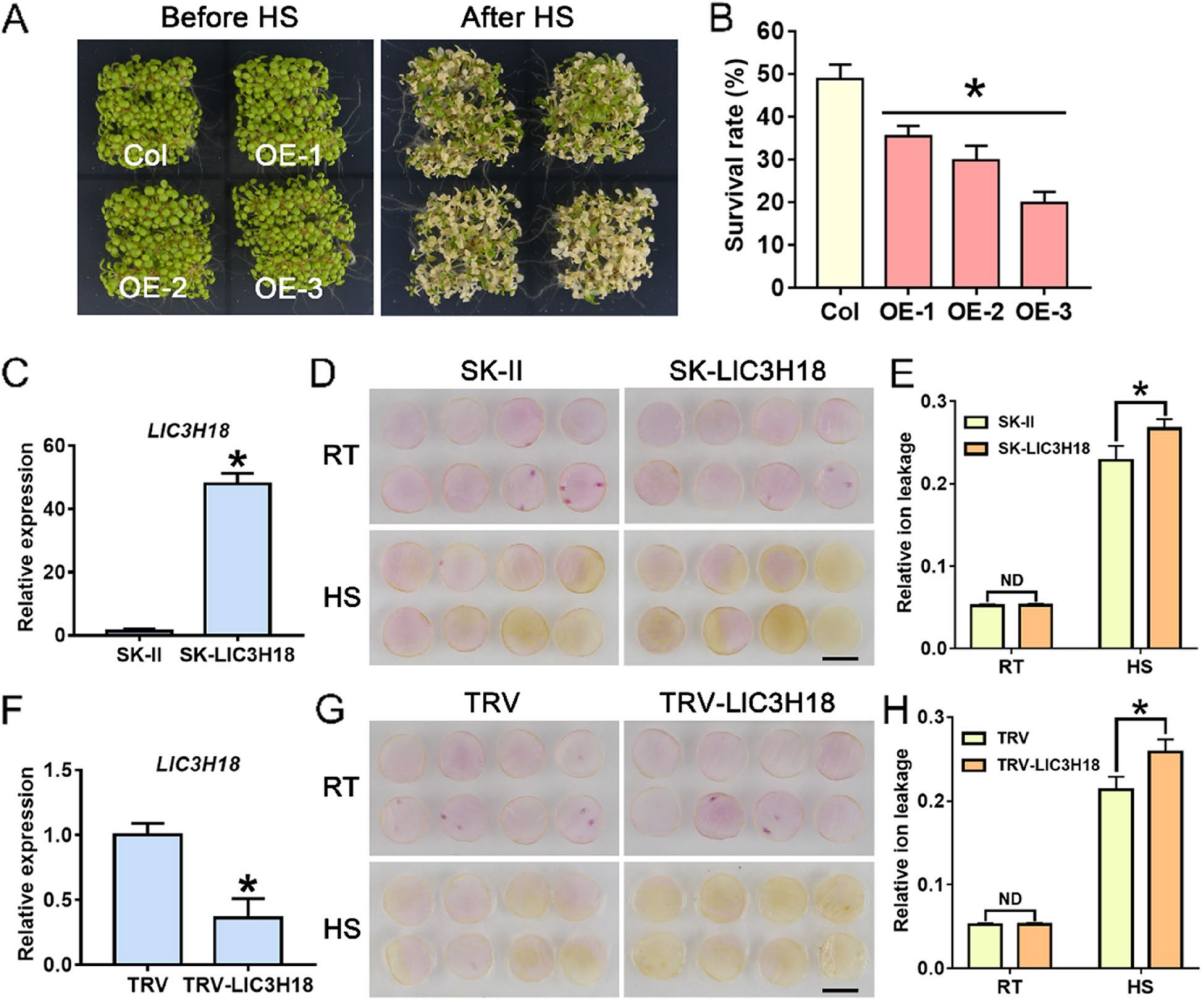
Thermotolerance analysis of *LlC3H18-*overexpressed and -silenced petal discs, and *LlC3H18*-overexpressed Arabidopsis plants. **A** The 5-d-old seedlings were directly exposed to 45℃ conditions for 1 h to detect their thermotolerance ability; the figure is a photo image taken after 7 days of recovery at 22℃. **B** The survival rate, measured after 7 days of heat stress (HS). Bars are the mean ± SD of three independent experiments (Student’s *t*-test, *P < 0.05). **C** Detection of *LlC3H18* expression in the *LlC3H18*-overexpressed petal discs. Data are presented as the mean ± SD of three replicates (Student’s *t*-test, * P < 0.05). **D** Phenotypes of lily petal discs under room temperature conditions (RT, 22 °C) and after exposure to heat stress (HS, 40 °C, 12 h). Representative image came from three experiments. Scale bar = 1 cm. **E** Relative ion leakage (%) of discs at 22 °C (RT) and after HS (40 °C, 12 h). Data are presented as the mean ± SD of three replicates (Student’s *t*-test, * *P* < 0.05; ND, no significant difference; the SK-LlC3H18 was compared with the SK-II control under the RT or HS condition, respectively. **F** Expression of *LlC3H18* in TRV-VIGS lily petals. Data are presented as the means ± SD of three replicates (Student’s *t*-test, * P < 0.05). **G** Phenotypes of lily petal discs at RT (22 °C) and after HS (40 °C, 12 h). Representative image based on three experiments. Scale bar = 1 cm. (H) Relative ion leakage (%) of discs at RT and after HS (40 °C, 12 h). Data are presented as the mean ± SD of three replicates (Student’s *t*-test, *P < 0.05; *ND* No significant difference, TRV-LlC3H18 was compared with the TRV-control under the RT or HS condition, respectively)

The seedlings of wild-type and overexpressing Arabidopsis plants were treated with HS to detect their thermotolerance (Fig. 5A). The results showed that overexpression of *LlC3H18* reduced the survival rate of transgenic seedlings after HS (Fig. 5B), indicating that LlC3H18 accumulation decreased their thermotolerance. Then we detected the expression of some heat-related genes in transgenic lines. It was revealed that overexpression of *LlC3H18* activated the expression of heat-related genes, *AtHSFA2*, *AtDREB2A*, *AtWRKY33*, *AtHSP22.0*, *AtHSP25.3*, and *AtGolS1* under normal conditions, but some of their expression (*AtHSFA2*, *AtDREB2A*, *AtHSP22.0*, *AtHSP25.3*, and *AtGolS1*) were decreased in one or two transgenic lines compared to the wild-type after HS treatment (Fig. S4), which suggested that *LlC3H18* overexpression might inhibit their expression under high temperature conditions, thereby causing reduced thermotolerance. At the same time, *LlC3H18* was also overexpressed in petal discs using the transient-expression system of lily (Fig. 5C). Followed by HS treatment, it was observed that *LlC3H18* overexpression accelerated the process of petal fading, and the fading of *LlC3H18-*overexpressed discs was stronger (Fig. 5D). In addition, under normal conditions, *LlC3H18* overexpression did not affect the relative ion leakage of petal discs, but after HS, the relative ion leakage of the overexpressed discs was higher than that of the controls (Fig. 5E). These results suggested that overexpression of *LlC3H18* limited the ability of lily cells to resist the damages of HS, and decreased the thermotolerance.

### Silencing of *LlC3H18* reduces thermotolerance in lily

The *LlC3H18* was silenced in lily petals by virus-induced gene silencing (VIGS), followed by thermotolerance assays (Fig. 5F). The results showed that silencing of *LlC3H18* promoted the fading of petals under HS conditions, suggesting that the damage caused by high temperature was aggravated in *LlC3H18*-silenced petal discs (Fig. 5G). Simultaneously, it was also observed that *LlC3H18* silencing did not affect the value of relative ion leakage of petal discs under normal conditions, but under HS, compared with the controls, the *LlC3H18*-silenced petal discs had a higher relative ion leakage (Fig. 5H), indicating that silencing of *LlC3H18* impaired the resistance of lily cells to high temperature and reduced their thermotolerance. On the other hand, the *atc3h18* homozygous Arabidopsis mutant (SALK_128806) was identified using PCR assays, and its thermotolerance was detected (Fig. S5). After HS, more mutant than wild-type seedlings died with a lower survive rate, which indicated *atc3h18* mutant was more susceptible to HS (Fig. S5). Then, the expression of some heat-related genes was quantified in *atc3h18*. Under normal conditions, *atc3h18* deficit did not affect the expression of the detected heat-related genes, but their expression was decreased in *atc3h18* compared to the wild-type after HS (Fig. S6). These results suggested that the appropriate expression of *C3H18* was crucial for establishing thermotolerance in Arabidopsis and lily.

### LlC3H18 binds to the promoter of *LlWRKY33* and activates its expression

**Fig. 6:**
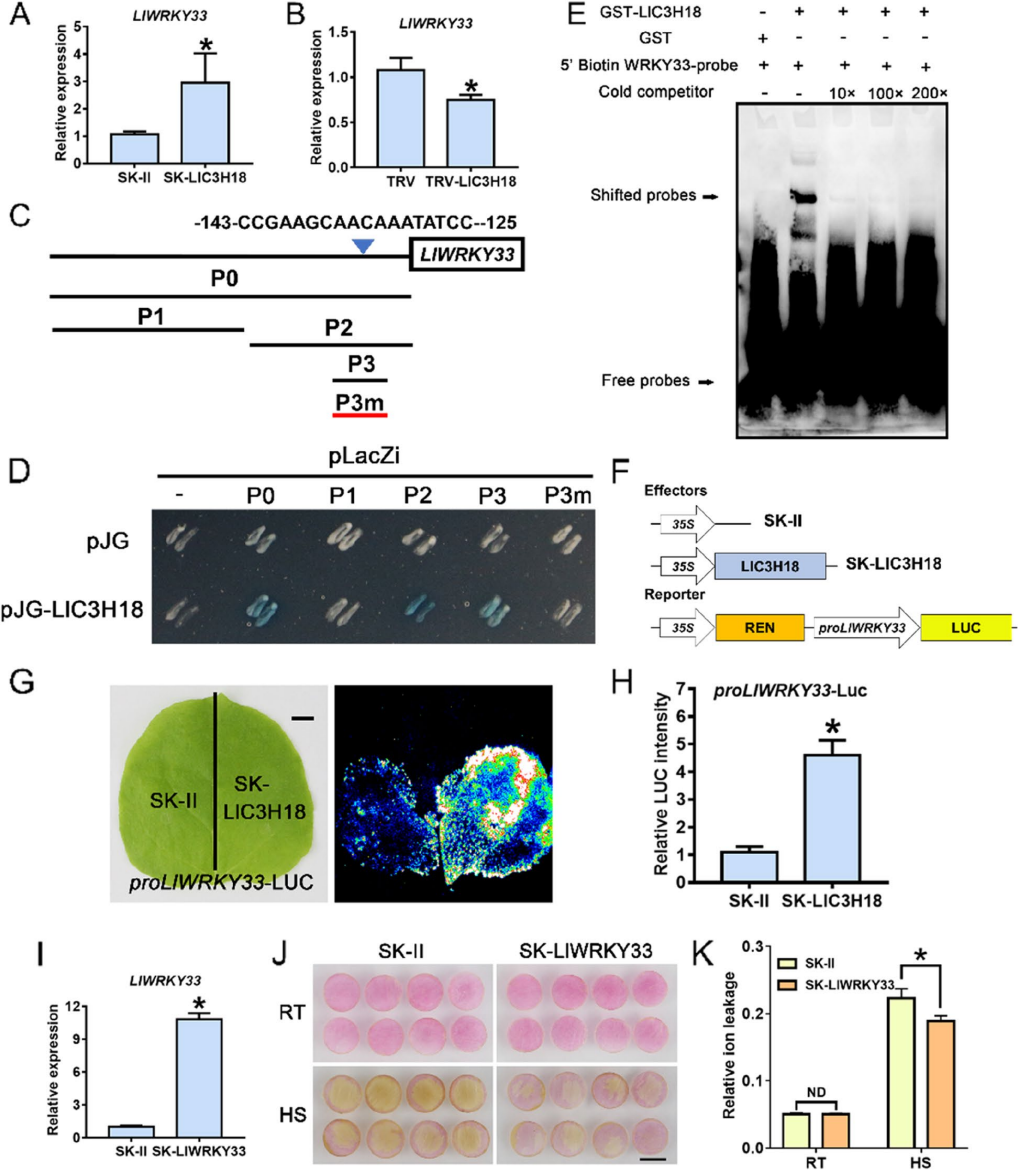
LlC3H18 binds the promoter of *LlWRKY33* and activates its expression. **A** Detection of the expression level of *LlWRKY33* in *LlC3H18*-overexpressed lily petals. Data are presented as the mean ± SD of three replicates (Student’s *t*-test, * *P* < 0.05). **B** Detection of the expression level of *LlWRKY33* in *LlC3H18*-silencing lily petals. Data are presented as the mean ± SD of three replicates (Student’s *t*-test, * *P* < 0.05). **C** Diagram of the *LlWRKY33* promoter. The W-box elements are marked with blue triangles. The truncated fragments used for the yeast one-hybrid (Y1H) assay are marked with black lines. The mutant fragment used for the Y1H assay is marked with a red line. **D** A Y1H assay for LlC3H18 and the promoter of *LlWRKY33*. Fragment activity was analyzed by a color change on Ura-/Trp-deficient SD medium following the addition of x-gal. One representative image based on three replicates. **E** An electrophoretic mobility shift assay (EMSA) of GST-LlC3H18 and the potential elements from the *LlWRKY33* promoter. One representative image based on three replicates. **F** Constructs used in the dual-luciferase reporter assay. **G** Detection of the LUC signal in tobacco leaves. One representative image based on three replicates. Scale bar = 1 cm. **H** Measurement of LUC intensity in the dual-luciferase reporter assay. Data are presented as means ± SD of three replicates (Student’s *t*-test, * *P* < 0.05). (I) Detection of *LlWRKY33* expression in the *LlWRKY33*-overexpressed petal discs. Data are presented as the mean ± SD of three replicates (Student’s *t*-test, * P < 0.05). (J) Phenotypes of lily petal discs under room temperature conditions (RT, 22 °C) and after exposure to heat stress (HS, 40 °C, 12 h). Representative image came from three experiments. Scale bar = 1 cm. (K) Relative ion leakage (%) of discs at 22 °C (RT) and after HS (40 °C, 12 h). Data are presented as the mean ± SD of three replicates (Student’s *t*-test, * *P* < 0.05; ND, no significant difference; the SK-LlWRKY33 was compared with the SK-II control under the RT or HS condition, respectively

Previous reports have demonstrated that WRKY33 plays an important role in the establishment of thermotolerance and resistance to *Botrytis cinerea*, and it is involved in the regulation of pathogenic response pathways as a target factor of multiple CCCH proteins, such as C3H14 (Birkenbihl et al. 2012; Li et al. 2011; Wang et al. 2020; Zheng et al. 2006). We first detected the expression of *AtWRKY33* in *LlC3H18*-overexpressing Arabidopsis plants, and it was found that the transgenic lines had a higher *AtWRKY33* expression than that of wild-type plants (Fig. S4). In addition, it was found that the expression of *LlWRKY33* in the *LlC3H18*-overexpressing petal discs was activated compared with the control discs (Fig. 5C, 6A); however, in the *LlC3H18*-silencing petal discs, the expression of *LlWRKY33* was evidently inhibited (Fig. 5F, 6B). Therefore, we speculated that LlWRKY33 may act as a downstream target of LlC3H18. Through a Y1H assay, it was observed that LlC3H18 directly bound to the promoter of *LlWRKY33* (33-P0) (Fig. 6C, D). Then, the *LlWRKY33* promoter was truncated into two fragments (33-P1 and 33-P2). The results of Y1H assay showed that LlC3H18 bound to the fragment 33-P2, but not the fragment 33-P1 (Fig. 6C, D). The fragment 33-P2 was further truncated into the fragment 33-P3, and LlC3H18 was found to bind the fragment 33-P3 (Fig. 6C, D). The core element of the 33-P3 fragment was mutated to form 33-P3m, and LlC3H18 could not bind to 33-P3m (Fig. 6C, D). The result of EMSA indicated that LlC3H18 could bind to the core element of 33-P3 in vitro (Fig. 6E; Table S1), suggesting that LlC3H18 was able to bind DNA element and directly bound to the promoter of *LlWRKY33*. The further dual-luciferase reporter assay showed that LlC3H18 activated the promoter activity of *LlWRKY33* (Fig. 6F-H). Therefore, these data suggested that LlC3H18 bound to the promoter of *LlWRKY33* and activate its expression. The role of LlWRKY33 was also detected by the transient overexpression system of lily petal discs (Fig. 6I). Same to the controls, overexpression of *LlWRKY33* did not affect the color and relative ion leakage of petals under normal conditions, but after HS, *LlWRKY33* overexpression reduced the fading of petal discs, and the relative ion leakage of them was also lower than the controls (Fig. 6J, K). These results showed that overexpression of *LlWRKY33* in lily improved the resistance of cells to HS.

### LlMYB305 binds to the promoter of *LlC3H18* and activates its expression

**Fig. 7:**
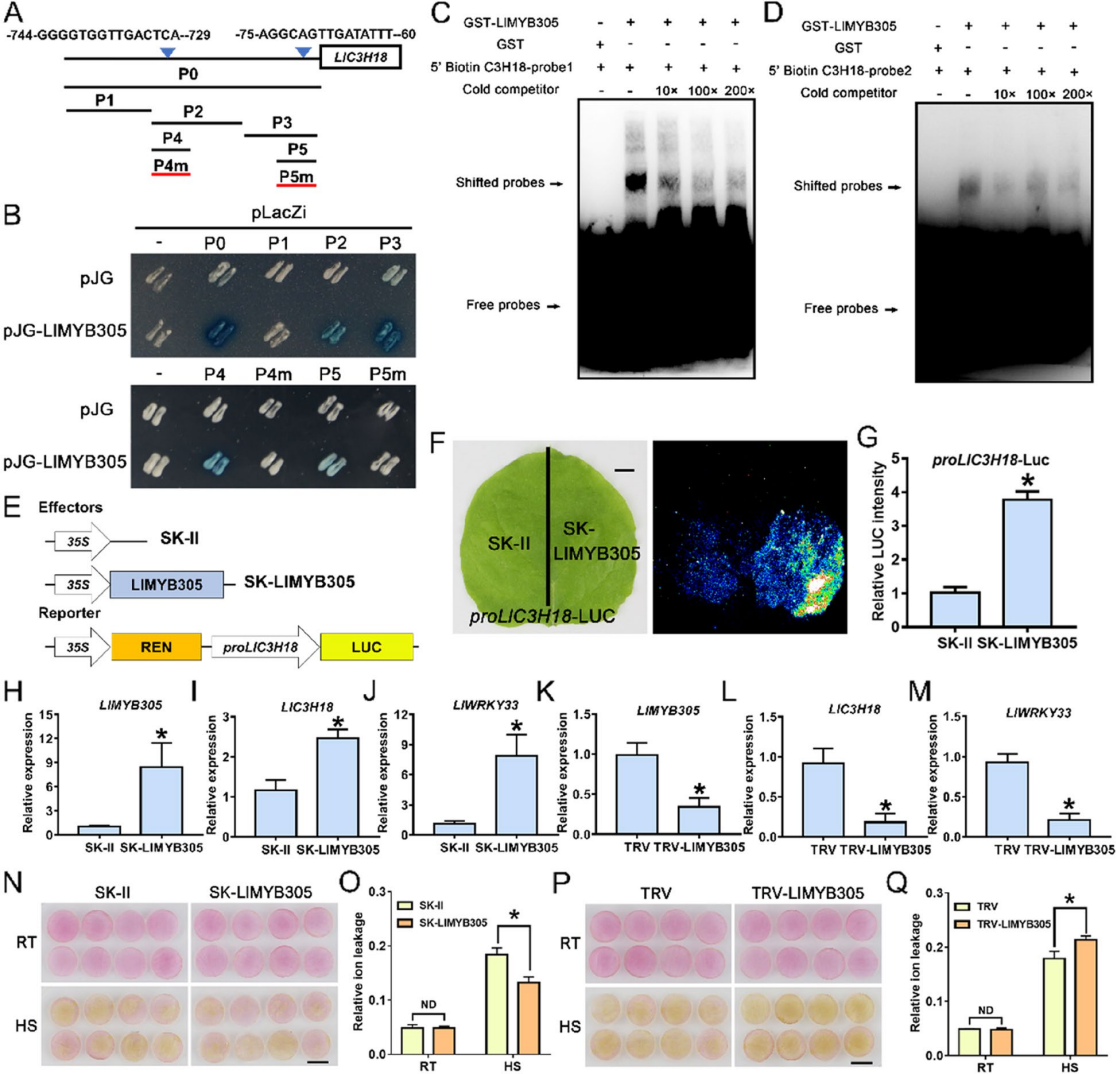
LlMYB305 binds the promoter of *LlC3H18* and activates its expression. (**A**) Diagram of the *LlC3H18* promoter. The W-box elements are marked with blue triangles. The truncated fragments used for the yeast one-hybrid (Y1H) assay are marked with black lines. The mutant fragment used for the Y1H assay is marked with a red line. (**B**) A Y1H assay for LlMYB305 and the promoter of *LlC3H18*. Fragment activity was analyzed by a color change on Ura-/Trp-deficient SD medium following the addition of x-gal. One representative image based on three replicates. (**C** and **D**) An electrophoretic mobility shift assay (EMSA) of GST-LlMYB305 and the potential elements from the *LlC3H18* promoter. The probe 1 and 2 came from the core sequence of P4 and P5 fragments, respectively. One representative image based on three replicates. (**E**) Constructs used in the dual-luciferase reporter assay. (**F**) Detection of the LUC signal in tobacco leaves. One representative image based on three replicates. Scale bar = 1 cm. (**G**) Measurement of LUC intensity in the dual-luciferase reporter assay. Data are presented as means ± SD of three replicates (Student’s *t*-test, * *P* < 0.05). (**H**-**J**) Detection of the expression level of *LlMYB305* (**H**), *LlC3H18* (**I**), and *LlWRKY33* (**J)** in *LlMYB305*-overexpressed lily petals. Data are presented as the mean ± SD of three replicates (Student’s *t*-test, * *P* < 0.05). (K-M) Detection of the expression level of *LlMYB305* (**K**), *LlC3H18* (**L**), and *LlWRKY33* (**M**) in *LlMYB305*-silencing lily petals. Data are presented as the mean ± SD of three replicates (Student’s *t*-test, * *P* < 0.05). (**N**) Phenotypes of lily petal discs under room temperature conditions (RT, 22 °C) and after exposure to heat stress (HS, 40 °C, 12 h). Representative image came from three experiments. Scale bar = 1 cm. (**O**) Relative ion leakage (%) of discs at 22 °C (RT) and after HS (40 °C, 12 h). Data are presented as the mean ± SD of three replicates (Student’s *t*-test, * *P* < 0.05; ND, no significant difference; the SK-LlMYB305 was compared with the SK-II control under the RT or HS condition, respectively. (**P**) Phenotypes of lily petal discs at RT (22 °C) and after HS (40 °C, 12 h). Representative image based on three experiments. Scale bar = 1 cm. (**Q**) Relative ion leakage (%) of discs at RT and after HS (40 °C, 12 h). Data are presented as the mean ± SD of three replicates (Student’s *t*-test, *P < 0.05; ND, no significant difference; TRV-LlMYB305 was compared with the TRV-control under the RT or HS condition, respectively)

The study of poplar has reported that PdC3H18 can participate in the formation of secondary cell walls as a downstream factor of PdMYB21 (Chai et al. 2014). Our previous study has shown that lily LlMYB305 is a MYB21 homology whose expression is activated by high temperature and plays a positive role in thermotolerance (Wu et al. 2021). In addition, MYB21 and C3H18 have been reported to participate in the same process of anther development (Cheng et al. 2009; Huang et al. 2020; Song et al. 2011; Xu et al. 2022). According to these results, we guessed that LlMYB305 might act as an upstream regulator of *LlC3H18*. Through a Y1H assay, it was revealed that LlMYB305 directly bound to the *LlC3H18* promoter (18-P0), and the truncation analysis of *LlC3H18* promoter showed that LlMYB305 bound to the fragments 18-P2 and 18-P3, but not to 18-P1 (Fig. 7A, B). Analysis of the 18-P2 and 18-P3 sequences showed that both them contained a conserved MYB-responsive element (MRE) (Fig. 7A). The fragments 18-P2 and 18-P3 were then truncated into 18-P4 and 18-P5, respectively. The result of Y1H assay reveled that LlMYB305 bound to them, but not to the mutant fragments 18-mP4 and 18-mP5 (Fig. 7A, B). The EMSA assay also indicated that LlMYB305 could bind to the MREs from the fragment 18-P4 (probe 1) and 18-P5 (probe 2) (Fig. 7C, D; Table S1). The further dual luciferase assay showed that LlMYB305 could activate the promoter activity of *LlC3H18* (Fig. 7E-G), suggesting that LlMYB305 might directly activate its expression. Besides, with transient overexpression of *LlMYB305* in lily petals, we found that the expression of *LlC3H18* and *LlWRKY33* in the *LlMYB305*-overexpressing petal discs was increased compared with the controls (Fig. 7H-J). However, silencing of *LlMYB305* in lily petals caused the decreased expression of *LlC3H18* and *LlWRKY33* (Fig. 7K-M). Compared with the control petal discs, discs overexpressing *LlMYB305* showed slower petal fading and lower relative ion leakage after high-temperature treatment (Fig. 7N, O), while silencing of *LlMYB305* showed opposite effects (Fig. 7P, Q), indicating that LlMYB305 positively regulates thermotolerance.

## Discussion

Plant CCCH proteins form a large family of regulatory proteins function in many aspects of plant growth, development, and environmental responses (Bogamuwa and Jang 2014; Han et al. 2021). In plants, the TZF proteins with two zinc-finger motifs usually account for the majority, and more studies of them have be performed, while there are few reports on non-TZF proteins. In this study, we identified a high-temperature differentially expressed non-TZF gene *LlC3H18* that plays a required role in thermotolerance (Fig. 1).

Similar to the protein structure of Arabidopsis AtC3H18 (Xu et al. 2022), LlC3H18 has a CCCH domain and two potential RBDs (Fig. S3). Under normal conditions or at the recovery period after HS, LlC3H18 distributed in the cytoplasm and nucleus, but under HS, LlC3H18 was localized in cytoplasmic foci, and co-localized with the PB and SB markers (Fig. 2A, B), suggesting that it might bind RNA and participate in assembly process of mRNP granules. Although most *CCCH* genes diverse in expression patterns and functions, all Arabidopsis RR-TZFs and another two TZFs (AtC3H14 and AtC3H15), and rice OsTZF1 can localize to cytoplasmic foci (Chai et al. 2015; Jan et al. 2013; Pomeranz et al. 2010a). Two non-TNF proteins of Arabidopsis, AtC3H18L and AtC3H18 can also locate in cytoplasmic foci after HS (Xu et al. 2020a; Xu et al. 2022). In Arabidopsis, multiple TZFs are localized in both the nucleus and cytoplasm foci, where they can function as both RNA-binding proteins and TFs, such as AtTZF1, AtC3H14, and AtC3H15 (Addepalli and Hunt 2008; Kim et al. 2014; Pomeranz et al. 2010b; Qu et al. 2014; Wang et al. 2020). There is a difference here, the localization of cytoplasmic foci of many TZFs is stable, while the localization of cytoplasmic foci of LlC3H18 was induced by high temperature (Fig. 2A), and after the release of HS, LlC3H18 repositioned in the cytoplasm and nucleus. Many TZF proteins, such as AtTZF1 and AtC3H14 of Arabidopsis, SlC3H39 of tomato, and OsC3H12 of rice, act as RNA-binding proteins, which can bind the conserved ARE at the 3’-UTR of mRNA to regulate their stability and translation efficiency (Deng et al. 2012; Kim et al. 2014; Pomeranz et al. 2010b; Qu et al. 2014; Xu et al. 2023), but whether non-TZF proteins bind to RNA is still unknown. In this study, we found that LlC3H18, as a non-TZF protein, can also bind to the typical ARE to affect the stability of mRNA (Fig. 2).

Under normal conditions or at the recovery period after HS, it was observed that part of LlC3H18 was located into the nucleus (Fig. 2A), implying that it might also function as a TF. In addition, LlC3H18 showed transactivation activity in both yeast and tobacco cells (Fig. 3), indicating that it may be able to directly activate the expression of target genes. AtC3H14 and AtC3H15 are mainly localized to cytoplasm foci, and a small part is localized in the nucleus, but they show transactivation activity and activate target genes (Chai et al. 2015). AtWRKY33 has been reported to be involved in the regulation of plant pathogen defense, and salt, flooding, and heat tolerances, which is a central regulator of these physiological processes (Jiang and Deyholos 2008; Krishnamurthy et al. 2020; Li et al. 2011; Liu et al. 2021; Liu et al. 2015; Zheng et al. 2006). AtC3H14 shows an ability to bind DNA element and acts as a direct regulator of *AtWRKY33* to activate its expression and participate in the establishment of resistance to *B. cinerea* (Wang et al. 2020). Similarly, we found that LlC3H18 bound to the DNA element from *LlWRKY33* promoter and activated its expression (Fig. 6). Overexpression of *LlC3H18* activated the expression of WRKY33 in lily and Arabidopsis, and silencing of *LlC3H18* decreased the expression of *LlWRKY33* (Fig. 6). These results suggested that LlC3H18 might act as a direct activator of *LlWRKY33*.

At room temperature, AtC3H18 could form mRNP granules in pollens and localize to cytoplasmic foci, but no similar phenomenon was observed in tobacco cells. However, under HS conditions, AtC3H18 can form mRNP granules in tobacco cells and localize to cytoplasmic foci, indicating the critical concentration of AtC3H18 forming mRNP granules in pollens is lower than that in tobacco cells (Xu et al. 2022). The localization of LlC3H18 also had a similar phenomenon; the localization of cytoplasmic foci of LlC3H18 was enabled by high temperature (Fig. 2A). In Chinese cabbage, the *C3H18* homologous genes *BcMF30a* and *BcMF30c* play an indispensable role in pollen fertility; overexpression or mutation of them leads to abnormal pollen development, indicating that proper expression of *BcMF30a* and *BcMF30c* is extremely important for normal pollen development (Xu et al. 2020b; Xu et al. 2020c). Similarly, *AtC3H18-*overexpressed Arabidopsis lines also show pollen abortion, which may be caused by affecting the assembly of mRNP granules (Xu et al. 2022). Our study found that overexpression of *LlC3H18* in lily and Arabidopsis would lead to the reduction of thermotolerance (Fig. 5). In *LlC3H18*-overexpressing plants, it was found that the expression of heat-responsive genes was activated at room temperature, indicating that LlC3H18 could act as a TF and activate HS response in the absence of high temperature; however, under HS, the induced expression of heat-responsive genes decreased, which might lead to a final decrease in thermotolerance (Fig. S4). We speculated that the excessive expression of *LlC3H18* resulted in the accumulation of LlC3H18 protein, which might destroy the normal assembly process of mRNP granules under HS conditions for leading to the damage of HS response and reducing the expression of heat-protective genes; the specific mechanism need to be clarified in the future. Interesting, silencing of *LlC3H18* in lily also led to the decrease of its thermotolerance (Fig. 5). Similarly, the *atc3h18* Arabidopsis mutant showed decreased thermotolerance as well. We found that the heat-induced expression of heat-responsive genes was reduced in *atc3h18* mutant (Fig. S6). After HS release, C3H18 was released from cytoplasmic foci and entered the nucleus to play a role as a trans-activator. The decrease or deletion of *C3H18* expression may disrupt the role of trans-activator of C3H18, which is detrimental to the maintenance of HS response. These results indicated that the appropriate expression of *C3H18* was crucial for establishing thermotolerance.

Meanwhile, many studies have also found that transgenic plants with overexpression of TZFs often show growth defects. For instance, the overexpression transgenic plants of *AtTZF1*, *4*, *5*, and *6* all exhibited compact and crinkled leaves, and some of homozygous overexpression plants of *AtTZF1* even showed lethal phenotype (Bogamuwa and Jang 2013; Lin et al. 2011). Overexpression of *AtC3H14* and *AtC3H15* led to dwarfing phenotypes and male sterility in Arabidopsis, respectively (Kim et al. 2014; Shi et al. 2015). We found that overexpression of *LlC3H18* also caused the growth defects of transgenic Arabidopsis plants (Fig. 4), which was very different with the growth phenotype of *AtC3H18*-overexpression, which did not cause any growth defects under normal conditions (Xu et al. 2022). It was speculated that LlC3H18 also showed nucleus-localization under normal conditions, which may activate some target genes to damage the growth of transgenic plants.

Arabidopsis *AtC3H18* is highly expressed in anthers, and the R2R3-MYB TFs AtMYB21 and AtMYB24 function key roles in anther development and thermotolerance (Cheng et al. 2009; Huang et al. 2017, 2020; Kumar and Chattopadhyay 2018; Mandaokar and Browse 2009; Song et al. 2011; Xu et al. 2022). Lily LlMYB305 is a MYB21/24 homology, and our previous study demonstrated that LlMYB305 is induced by high temperature (Wu et al. 2021). In this study, we demonstrated that LlMYB305 directly bound to the promoter of *LlC3H18* and activated its expression (Fig. 7). At the same time, we found that overexpression of *LlMYB305* improved the thermotolerance of lily, silencing of *LlMYB305* reduced its thermotolerance (Fig. 7); and LlMYB305 could activate the expression of *LlC3H18*, which did not damage thermotolerance as overexpression of *LlC3H18* (Fig. 5). It was speculated that LlMYB305, as an upstream regulatory factor, could coordinate the role of LlC3H18 by simultaneously activating other factors, thereby ensuring that LlC3H18 played a role within an appropriate range. In poplar, PdMYB3 and PdMYB21 regulate the specific expression of *PdC3H17* and *PdC3H18* to participate in the formation of secondary cell walls (Chai et al. 2014), suggesting that MYB TFs and CCCH-type proteins may link with a conserved regulatory mechanism.

**Fig. 8:**
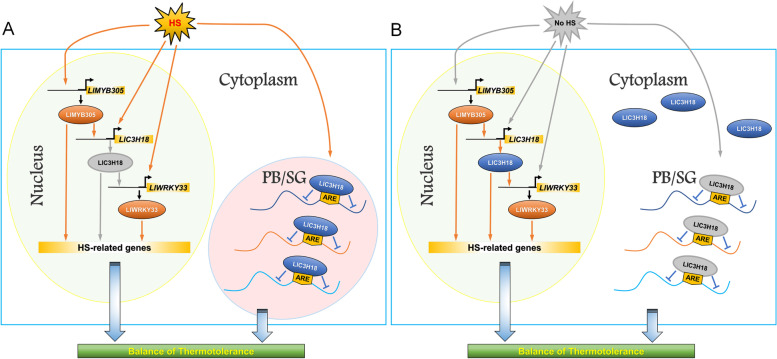
A simple working model of the LlC3H18-mediated regulatory mechanism in lily in response to heat stress. **A** Under HS conditions, *LlC3H18* is a heat-inducible *CCCH* gene, which can be directly activated by LlMYB305; LlC3H18 locates in the cytoplasm foci and acts as RNA binding protein to form mRNP granules, thus balancing the thermotolerance. **B** At the recovery period without HS, LlC3H18 can be transformed from cytoplasm foci to localize in the nucleus, which promotes it to act as a trans-activator, directly activating the expression of *LlWRKY33*, thus forming a heat-inducible LlMYB305-LlC3H18-LlWRKY33 regulatory module, and sustaining the heat stress response. The gray arrow indicates a closed state, while the yellow arrow indicates a working state. *HS* Heat stress, *PB* Processing body, *SG* Stress granule

In conclusion, our study showed that LlC3H18 was a heat-inducible CCCH-type protein, and LlMYB305 could act as its upstream factor to activate its expression and participate in the establishment of thermotolerance; without HS, LlC3H18 could localize in the nucleus and acted as a trans-activator to stimulate the expression of *LlWRKY33*; in addition, under HS conditions, LlC3H18 could also play a role of RNA-binding protein, form mRNP granules to participate in the regulation of thermotolerance (Fig. 8). Based on these results, we speculate that there may be a LlMYB305-LlC3H18-LlWRKY33 regulatory module involved in the establishment of thermotolerance in lily.

## Methods

### Plant materials and growth conditions

The *Lilium* Oriental hybrid ‘Sorbonne’ and *L. longiflorum* ‘White heaven’ were used as the experimental materials in this study. The ‘Sorbonne’ was planted in soil and grown in a greenhouse with day/night temperatures of 22/16 °C. Sterile tissue-cultured plantlets of ‘White heaven’ were cultured in a standard culture room at 22 °C with a light–dark cycle of 16 h/8 h. *Arabidopsis thaliana* Col-0 and *Nicotiana benthamiana* (tobacco) seeds were sown in MS medium, and 10 days after germination, the seedlings were transplanted into nursery pots and grown in a standard greenhouse (22/16 °C) with 16-h/8-h light/dark photoperiod.

### Isolation of *LlC3H18 *gene, *LlC3H18* and *LlWRKY33* promoters from ‘White heaven’

Two-week-old lily plants were treated with HS at 37 °C for 1 h, the leaves were collected, and total RNA was extracted using RNAprep Pure Kit (Tiangen, China), followed by M-MLV reverse transcriptase (Vazyme, China) and Oligo dT primer to synthesize cDNA. According to the transcriptome data, the specific primers were designed to amplify the open reading frame (ORF) of *LlC3H18* (Table S2). The promoters of *LlC3H18* and *LlWRKY33* were isolated and cloned with the method of Hi-tail PCR (Liu and Chen 2007) from lily ‘White heaven’; the 1184-bp upstream fragment from ATG of *LlC3H18*, and the 521-bp upstream fragment from ATG of *LlWRKY33* were isolated and identified, respectively.

### Phylogenetic tree analysis and prediction of conserved protein domains

Phylogenetic tree analysis of LlC3H18 and its homologous proteins was performed by MEGA 7.0 software using the neighbor-joining method (n = 1000). Multiple alignment analysis of C3H18 amino acid sequences from different species was performed using ClustalW 2.0 and BioEdit 7.0 softwares.

### Promoter activity analysis of *LlC3H18*

The *LlC3H18* promoter was cloned into pGreenII0800-LUC (Hellens et al. 2005) and pCAMBIA1391-GUS (Abcam, USA). The reconstructed pGreenII0800-LUC-*proLlC3H18* was introduced into *Agrobacterium tumefaciens* strain GV3101 (psoup). A mixed bacterial solution was infiltrated into tobacco leaves for the activity assay. After 48 h, the infiltrated leaves were treated with HS at 37 °C for 3 h, then they were removed to detect the LUC signal. The reconstructed pCAMBIA1391-GUS-*proLlC3H18* was introduced into *A. tumefaciens strain* GV3101. The GUS-reporter gene was stably transformed into Arabidopsis and transiently transformed into lily petal discs. The transgenic Arabidopsis seedlings and the lily petal discs were treated with HS at 37 °C for 3 h, and then, they were sampled for GUS assay. All primers used for plasmid construct are listed in the Table S3.

### The transcriptional activity assay of LlC3H18

The ORF of *LlC3H18* was cloned into pGBKT7 (BD; Clontech, Japan) to generate BD-LlC3H18 protein. The plasmid-transformed yeast AH109 cell was used for transcriptional activity analysis; yeast containing GAL4 and BD were used as positive and negative controls, respectively. After 3 days of culture at 30 °C, the positive clones were selected and transferred to -Trp-His-deficient SD medium for identification of transcriptional activity. The ORF of *LlC3H18* was cloned into the vector pEAQ (Sainsbury et al. 2009) to generate a BD fusion protein as effector. The 5 × GAL4 UAS element and the mini 35S promoter were fused and cloned into the vector pGreenII0800-LUC to construct the reporter vector. These reconstituted vectors were respectively introduced into *A. tumefaciens* GV3101 (psoup). The bacterial solution was resuspended, mixed according to the proportion, and then the tobacco leaves were injected. Under normal growth conditions, after culturing for 60 h, the injected leaves were cut to detect the LUC signal and the LUC intensity was also determined.

### Subcellular localization analysis of LlC3H18

The ORF of *LlC3H18* was cloned into pCAMBIA1300-N-GFP (Abcam, USA) vector to generate GFP-LlC3H18 fusion protein, and the reconstituted vector were introduced into *A. tumefaciens* GV3101, respectively. The different constructs were expressed in tobacco leaves, the mCherry-DCP2, mCherry-PABP8, and RFP-NLS were used as the PB, SG, and nucleus marker, respectively, and fluorescence signals were checked under confocal microscopy (Zeiss, Jena, Germany).

### Heat treatment and gene expression analysis of lily

The robust tissue-cultured ‘White heaven’ seedlings with the same size were selected for gene expression analysis. For HS, lily plantlets were incubated at 37 °C for different lengths of time (0, 0.5, 1, 3, 6, 12 h). After HS finished, the leaves were collected for extracting total RNA. The RNA reverse transcribed into cDNA with a HiScript II kit (Vazyme, China), and the expression of *LlC3H18* were detected by real-time quantitative PCR (RT-qPCR) with the 2^−ΔΔCT^ method (Livak and Schmittgen 2001; Schmittgen and Livak 2008). Lily 18S rRNA was used as an internal reference gene (Table S4).

### Yeast one-hybrid assay

The test and mutant fragments of the promoters of *LlC3H18* and *LlWRKY33* were cloned into pLacZi (Clontech, Japan) vectors. The ORFs of *LlMYB305* and *LlC3H18* were inserted into a pJG (Clontech, Japan) vector, respectively. The corresponding vectors were co-transformed into yeast EGY48. Successful transformants were selected by growing on -Trp-Ura deficit SD media for 3 days at 30℃. Binding was investigated using color analysis on SD media containing 80 mg L^−1^ x-gal.

### Electrophoretic mobility shift assay (EMSA)

The ORFs of *LlC3H18* and *LlMYB305* were separately cloned into pGEX-4 T-1 (GE Healthcare, USA) to generate the GST fusion proteins. The fusion proteins were induced in *E. coli* BL21 by adding isopropyl-β-D-1-thiogalactopyranoside (200 mM, IPTG); The recombinant GST-LlC3H18 and GST-LlMYB305 proteins was purified by GST protein purification kit, and detected by SDS–polyacrylamide electrophoresis. The EMSA probe was synthesized with 5’ biotin-labeled. Binding reactions were incubated at room temperature for 20 min and then separated using electrophoresis through a 6% (40:1 acrylamide:bis-acrylamide) native gel at 4 °C. The EMSA analysis was performed using the Light Shift Chemiluminescence EMSA Kit (ThermoFisher, New York, USA).

### Dual-luciferase reporter assay

The ORFs of *LlC3H18* and *LlMYB305* were cloned into pGreenII62-SK (SK-II) (Hellens et al. 2005) to generate effector vectors, respectively. The *LlC3H18* and *LlWRKY33* promoters were cloned into pGreenII0800-LUC to generate the reporter vectors. The empty vectors were used as the negative control. The vectors were introduced into *A. tumefaciens* strain GV3101 (psoup). A mixed bacterial solution was infiltrated into tobacco leaves for the dual-luciferase reporter assay. After 48 h, the infiltrated leaves were removed, and the LUC signal was detected and measured.

### ARE-binding assay

For DNA constructs used in mRNA-binding assays, GFP-ARE was made by ligating the ARE (5’-TTATTTATTATTTATTTATTATTTATTTATTATTTATTTATTA-3’) to the end of the GFP coding region, subcloning to pCAMBIA1300-GFP vector. GFP-MutG was generated following the same procedures except the ARE region was replaced by the MutG (5’-TTGTTTGTTGTTTGTTTGTTGTTTGTTTGTTGTTTGTTTGTTA-3’). The LlC3H18 was generated by PCR and then transferring it in pGreenII62-SK (Hellens et al. 2005) vector. These reconstituted vectors were introduced into *A. tumefaciens* GV3101, respectively. The mixed bacterial solutions were suspended and injected in tobacco leaves. After 48 h, the leaves were removed and the intensity of GFP fluorescence was observed.

### Transient overexpression in lily petals

The bacterial solutions of pGreenII62-SK and pGreenII62-SK-LlC3H18/LlWRKY33/LlMYB305 were collected by centrifugation and resuspended in the infiltration buffer (10 mM MgCl_2_, 200 mM acetosyringine, 10 mM MES, pH 5.6) and placed in the dark at 22℃ for 5 h before vacuum infiltration. The 10-cm length of unopened ‘Sorbonne’ flower buds was selected, and the inner petals were used to obscure the 1-cm-diameter discs with a hole-puncher (Wu et al. 2022a; Wu et al. 2023; Wu et al. 2022b). Under the vacuum condition of -0.7 MPa, the bacterial solution was infiltrated into these petal discs. Then, the discs were washed with sterile water and placed on an agar plate (0.4%), and cultivated in the dark at 22 °C for 96 h. For HS treatment, the discs were treated at 40 °C for 12 h, then harvested immediately, and their relative ion leakage determined (Wu et al. 2023).

### Virus-induced gene silencing (VIGS) in lily petals

A 300-bp fragment of *LlC3H18* or *LlMYB305* was obtained by PCR amplification and cloned into pTRV2 to generate the pTRV2-LlC3H18 and pTRV2-LlMYB305 vector, respectively. Then, pTRV1, pTRV2, and pTRV2-LlC3H18 or pTRV2-LlMYB305 were transformed into *A. tumefaciens* GV3101, respectively. The bacteria of TRV1 and TRV2 were resuspended and mixed in proportion, and the petal discs were vacuumed as described above. After 5 days, the discs were treated with HS, then the discs were harvested to measure relative ion leakage.

### Stable transformation of Arabidopsis

The *LlC3H18* ORF was inserted into pCAMBIA1300, the *LlC3H18* promoter was inserted into pCAMBIA1391, and then the reconstructed vectors were transformed into *A. tumefaciens* GV3101, respectively. For transformation, the floral-dip method was used with 5-week-old Arabidopsis plants. Through resistance screening and RT-PCR identification, three transgenic lines were selected for subsequent experiments.

### Thermotolerance test of transgenic plants

Arabidopsis seeds were sterilized and sown on MS medium, vernalized for 3 days at 4 °C in the dark, and then transferred to a standard culture room at 22 °C (16 h light/8 h dark). The 5-day-old Arabidopsis seedlings were treated with HS in a constant temperature incubator, and recovery at 22 °C for 7 days, and the survival rate was recorded. The 5-day-old wild-type and transgenic Arabidopsis seedlings were collected for expression analysis of heat-responsive genes. Arabidopsis *AtActin2* was used as internal reference gene for RT-qPCR analysis, and the relative levels of gene expression were calculated using the 2^−ΔΔCT^ method (Livak and Schmittgen 2001; Schmittgen and Livak 2008).

### Supplementary Information

**Additional file 1: Supplementary Table S1. **The promoter sequences are used for EMSA assay.**Supplementary Table S2. **Primers of LlC3H18 isolation.**Supplementary Table S3. **Primers used for vector reconstruction.**Supplementary Table S4. **RT-qPCR primers.**Supplementary Table S5. **Primers used for atc3h18 mutant identification.

**Additional file 2: Supplementary Figure S1. **Heat map of the expression of CCCH-type genes in lily leaves with heat stress.** Supplementary Figure S2. **Phylogenetic analysis of LlC3H18 and CCCH-type proteins of Arabidopsis.**Supplementary Figure S3. **Sequence analysis of LlC3H18.**Supplementary Figure S4. **Expression analysis of heat-related genes in the wild-type and LlC3H18 transgenic plants under normal and heat stress conditions.**Supplementary Figure S5. **Identified T-DNA insertion atc3h18 mutant and detection its thermotolerance. **Supplementary Figure S6. **Expression analysis of heat-related genes in the wild-type and atc3h18 mutant plants under normal and heat stress conditions.

## Data Availability

The authors confirm that all data in this study are included in this published article (and its supplementary information file).

## Abbreviations

ARE: AU-rich element
CCCH: Three conserved cysteine residues and one histidine residue
EMSA: Electrophoretic mobility shift assay
GFP: Green fluorescent protein
HS: Heat stress
LUC: Luciferase
mRNP: Messenger ribonucleoprotein
OE: Overexpression
ORF: Open reading frame
PB: Processing body
PCR: Polymerase chain reaction
SG: Stress granule
TF: Transcription factor
TRV: Tobacco rattle virus
TZF: CCCH-type zinc finger
VIGS: Virus-induced gene silencing
Y1H: Yeast one-hybrid
