# Supplementary material for: A LlMYB305-LlC3H18-LlWRKY33 module regulates thermotolerance in lily
Source: Mol Hortic. 2023 Aug 17;3:15. doi: 10.1186/s43897-023-00064-1 (PMC10514960; doi:10.1186/s43897-023-00064-1)
Supplement: Supplementary file 1 — Additional file 1: Supplementary Table S1. The promoter sequences are used for EMSA assay.Supplementary Table S2. Primers of LlC3H18 isolation.Supplementary Table S3. Primers used for vector reconstruction.Supplementary Table S4. RT-qPCR primers.Supplementary Table S5. Primers used for atc3h18 mutant identification. [file 43897_2023_64_MOESM1_ESM.docx]

**Supplementary Table S1.** The promoter sequences are used for EMSA assay.

| **Promoter fragments** | **Sequences** |
| --- | --- |
| *ProLlC3H18*-probe1 | ATAGGGTACCTAATCTGAAAATGAGCACATGGGGTGGTTGACTCATCAAA |
| *ProLlC3H18*-probe2 | GTCTATTCAGGAGGCAGTTGATATTTAGCTGATATTTTTC |
| *ProLlWRKY33*-probe | CAATTTCGCCTCCTTCTCCGAAGCAACAAATATCCATTTCATACGTGAGT |

**Supplementary Table S2.** Primers of *LlC3H18* isolation.

| **Primer name** | **Sequences** |
| --- | --- |
| *LlC3H18-*ORF-F | 5’-ATGATGTGCACTATGGGAGGGGGA-3’ |
| *LlC3H18-*ORF-R | 5’-CTATGTTACTGTAAAGATGCTGCTTGT-3’ |

**Supplementary Table S3.** Primers used for vector reconstruction.

| **Plasmid name** | **Vectors** | **Primer sequences** |
| --- | --- | --- |
| pCAMBIA1300- GFP | GFP-LlC3H18 | 5’- CTCGGCATGGACGAGCTGTACAAGGTCGAC  ATGATGTGCACTATGGGAGGGGGA -3’  5’- ATGTTTGAACGATCGGGGAAATTCGAGCTC  CTATGTTACTGTAAAGATGCTGCTTGT -3’ |
| pGBKT7 | BD-LlC3H18 | 5’-GACCTGCATATGGCCATGGAGGCCGAATTC ATGATGTGCACTATGGGAGGGGGA-3’  5’-GGGTTATGCTAGTTATGCGGCCGCTGCAG CTATGTTACTGTAAAGATGCTGCTTGT-3’ |
| pEAQ-BD | pBD-LlC3H18 | 5’-AGACAGTTGACTGTATCGCCGACCGGT ATGATGTGCACTATGGGAGGGGGA-3’  5’-ATTTAATGAAACCAGAGTTAAAGGCCT CTATGTTACTGTAAAGATGCTGCTTGT-3’ |
| pJG | pJG-LlC3H18 | 5’-GATGTGCCAGATTATGCCTCTCCCGAATTC ATGATGTGCACTATGGGAGGGGGA-3’  5’-CTCTGGCGAAGAAGTCCAAAGCTTCTCGAG CTATGTTACTGTAAAGATGCTGCTTGT-3’ |
| pJG | pJG-LlMYB305 | 5’-GATGTGCCAGATTATGCCTCTCCCGAATTC ATGGACAAGAGAGTGATCCCTGGT-3’  5’-CTCTGGCGAAGAAGTCCAAAGCTTCTCGAG TTAGTCTCCATTGAAAGACTGCATAGC-3’ |
| pLacZi | pLacZi-LlC3H18 | 5’-TTTCCTTTGATATTGGATCGGAATTC  TCATGGGGTGTGTCTCCCCTAAAT-3’  5’-TATACATACAGAGCACATGCCTCGAG  GCAGAGAAAGGAATTTCTTCTCAA-3’ |
| pLacZi | pLacZi-LlWRKY33 | 5’-TTTCCTTTGATATTGGATCGGAATTC  GTAAGAGAAGGAGAATATGCTTTC-3’  5’-TATACATACAGAGCACATGCCTCGAG  GGGAGGGGAAGAGGATGGGAAG-3’ |
| pGreenII 0800-SK | SK-LlC3H18 | 5’-GAGCTCCACCGCGGTGGCGGCCGCTCTAGA  ATGATGTGCACTATGGGAGGGGGA-3’  5’-GACGGTATCGATAAGCTTGATATCGAATTC  CTATGTTACTGTAAAGATGCTGCTTGT-3’ |
| pGreenII 0800-SK | SK-LlMYB305 | 5’-GAGCTCCACCGCGGTGGCGGCCGCTCTAGA ATGGACAAGAGAGTGATCCCTGGT-3’  5’-GACGGTATCGATAAGCTTGATATCGAATTC  TTAGTCTCCATTGAAAGACTGCATAGC-3’ |
| pGreenII 0800-SK | SK-LlWRKY33 | 5’-GAGCTCCACCGCGGTGGCGGCCGCTCTAGA ATGGCCTCCTCCACCGGAAGCT-3’  5’-GACGGTATCGATAAGCTTGATATCGAATTC  CTAGGCCAGCCATGAGTCTAGAA-3’ |
| pGreenII 0800-LUC | *ProLlC3H18*-Luc | 5’-GGCGAATTGGGTACCGGGCCCCCCCTCGAG TCATGGGGTGTGTCTCCCCTAAAT-3’  5’-GGTGGCGGCCGCTCTAGAACTAGTGGATCC GCAGAGAAAGGAATTTCTTCTCAA-3’ |
| pGreenII 0800-LUC | *ProLlWRKY33*-Luc | 5’-GGCGAATTGGGTACCGGGCCCCCCCTCGAG  GTAAGAGAAGGAGAATATGCTTTC-3’  5’-GGTGGCGGCCGCTCTAGAACTAGTGGATCC  GGGAGGGGAAGAGGATGGGAAG-3’ |
| pCAMBIA1391-GUS | *ProLlC3H18*-GUS | 5’-TTGGGCCCGGCGCGCCAAGCTT TCATGGGGTGTGTCTCCCCTAAAT-3’  5’-GTGGACTCCTCTTAGAATTCCCGGG  GCAGAGAAAGGAATTTCTTCTCAA-3’ |
| pTRV2 | pTRV2-LlC3H18 | 5’- TCTGTGAGTAAGGTTACCGAATTCTCTAGA  ATCGTGAGTAGAATTCATAGGGTGGA-3’  5’- CCGGGCCTCGAGACGCGTGAGCTCGGTACC  GATTGTCAATTGAAGCATGTCGGTCA-3’ |
| pTRV2 | pTRV2-LlMYB305 | 5’- TGTGAGTAAGGTTACCGAATTCTCTAGA  TGGATCTTCACTCCAGATGGGGA -3’  5’- GGGCCTCGAGACGCGTGAGCTCGGTACC  TATCGTTCGATTCAGTTGGAAAGG-3’ |
| pET32a | His-LlC3H18 | 5’- AAGGCCATGGCTGATATCGGATCCGAATTC  ATGATGTGCACTATGGGAGGGGGA-3’  5’- ATCTCAGTGGTGGTGGTGGTGGTGCTCGAG  TGTTACTGTAAAGATGCTGCTTGT-3’ |
| pGEX-4T-1 | GST-LlMYB305 | 5’-GATCTGGTTCCGCGTGGATCCCCGGAATTC  ATGGACAAGAGAGTGATCCCTGGT-3’  5’-CGTCAGTCAGTCACGATGCGGCCGCTCGAG  TTAGTCTCCATTGAAAGACTGCATAGC-3’ |
| pGEX-4T-1 | GST-LlC3H18 | 5’-GATCTGGTTCCGCGTGGATCCCCGGAATTC  ATGATGTGCACTATGGGAGGGGGA-3’  5’-CGTCAGTCAGTCACGATGCGGCCGCTCGAG  CTATGTTACTGTAAAGATGCTGCTTGT-3’ |

**Supplementary Table S4.** RT-qPCR primers.

| **Gene name** | **Gene ID** | **Sequences** |
| --- | --- | --- |
| *LlC3H18* |  | 5’-TCGCCCAACAGCCATATTTC-3’  5’-GCGAGCCACTGTTTAGAACC-3’ |
| *LlMYB305* |  | 5’-GCAGCTACCAGGGAGGACAGACA-3’  5’-ACTGCATAGCCCAGAACTCATCGTC-3’ |
| *LlWRKY33* |  | 5’-CGGATGTTGCAGCAGAACCAAGGGATG-3’  5’-AGGCCAGCCATGAGTCTAGAAGCAAGT-3’ |
| *18S rRNA* |  | 5’-AGTTGGTGGAGCGATTTGTCT-3’  5’-CCTGTTATTGCCTCAAACTTCC-3’ |
| *AtHSFA2* | *At2g26150* | 5’-GTGTTGAGGTTGGGCAATACG-3’  5’-TTGCTGTTGCCTCAACCTAACTAC-3’ |
| *AtDREB2A* | *At5g05410* | 5’-AGGGTCGAAGAAGGGTTGTATG-3’  5’-GGGAAAGTACCAAGCCAAAGC-3’ |
| *AtMBF1c* | *At3g24500* | 5’-GACGATGCCGAGCAGATACC-3’  5’-TTTCGGATCGCGTAGGTCTT-3’ |
| *AtGolS1* | *At2g47180* | 5’-AGCCGTTCATCACCGCTCTTAC-3’  5’-ACTCCTGGCAACATTCAAGCAG-3’ |
| *AtWRKY33* | *At2g38470* | 5’-GTGATATTGACATTCTTGACGA-3’  5’-GATGGTTGTGCACTTGTAGTA-3’ |
| *AtHSP25.3* | *At5g18340* | 5’-GATCAAGATGCGTTTCGACAT-3’  5’-TTCTACAGAGATTTTGACGTCTTCTT-3’ |
| *AtHSP22.0* | *At4g10250* | 5’-ACTACTCCAGGCAGCTTGCTA-3’  5’-CTTGAATGGATCAGGGAACC-3’ |
| *AtHSP70b* | *At1g16030* | 5’-TGCACGATGTTGTTCTGGTT-3’  5’-GCAAAAGCTGTTGAATTTTCG-3’ |
| *AtActin2* | *At3g18780* | 5’-TCCCTCAGCACATTCCAGCAGAT-3’  5’-AACGATTCCTGGACCTGCCTCATC-3’ |

**Supplementary Table S5.** Primers used for *atc3h18* mutant identification.

| **Primer name** | **Sequences** |
| --- | --- |
| LP | 5’-CTAAAAGTCTCCATGCCATCG-3’ |
| RP | 5’-CTCGCTTGAAGAACACGATTC-3’ |
| BP | 5’-ATTTTGCCGATTTCGGAAC-3’ |
