# Supplementary material for: A LlMYB305-LlC3H18-LlWRKY33 module regulates thermotolerance in lily
Source: Mol Hortic. 2023 Aug 17;3:15. doi: 10.1186/s43897-023-00064-1 (PMC10514960; doi:10.1186/s43897-023-00064-1)
Supplement: Supplementary file 2 — Additional file 2: Supplementary Figure S1. Heat map of the expression of CCCH-type genes in lily leaves with heat stress. Supplementary Figure S2. Phylogenetic analysis of LlC3H18 and CCCH-type proteins of Arabidopsis.Supplementary Figure S3. Sequence analysis of LlC3H18.Supplementary Figure S4. Expression analysis of heat-related genes in the wild-type and LlC3H18 transgenic plants under normal and heat stress conditions.Supplementary Figure S5. Identified T-DNA insertion atc3h18 mutant and detection its thermotolerance. Supplementary Figure S6. Expression analysis of heat-related genes in the wild-type and atc3h18 mutant plants under normal and heat stress conditions. [file 43897_2023_64_MOESM2_ESM.docx]

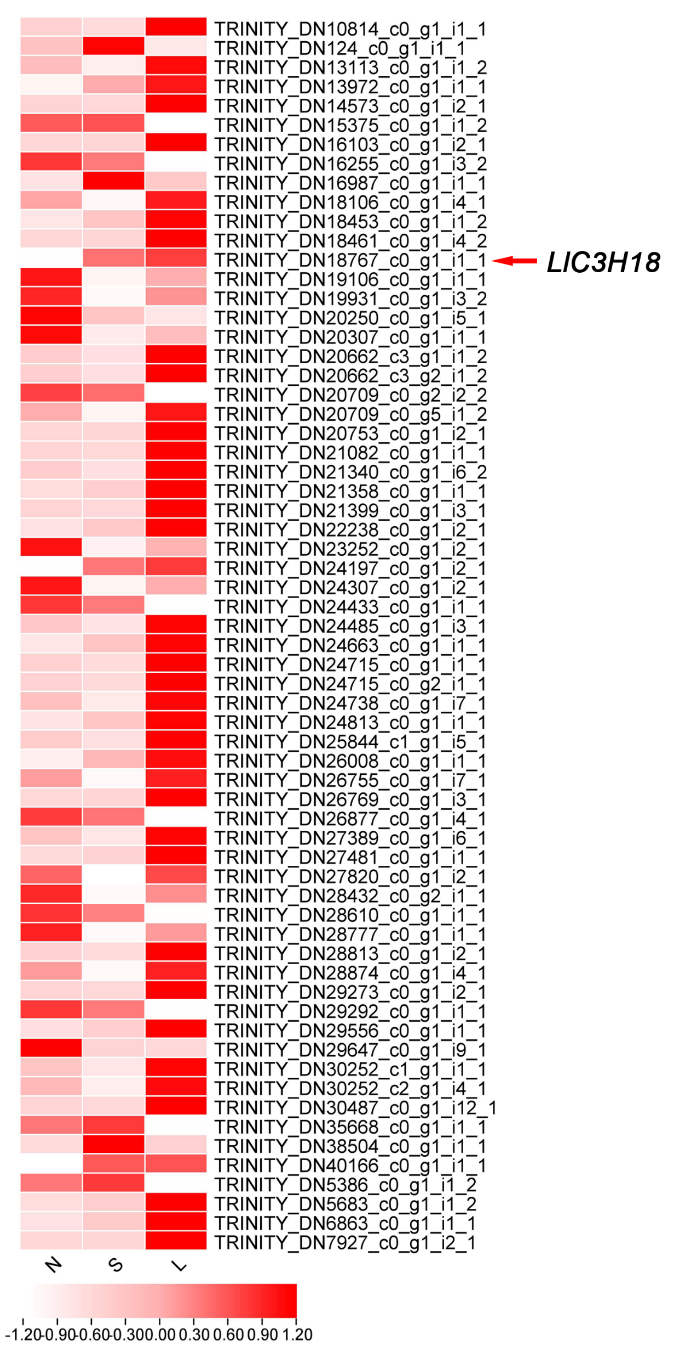


**Fig. S1** Heat map of the expression of *CCCH-*type genes in lily leaves with heat stress. Heat map visualizing the expression pattern of *CCCH-*type genes based on the heat stress transcriptome of lily (*Lilium longiflorum* ‘White heaven’) leaves, the red arrow indicates *LlC3H18*. N: normal condition (22℃); S: 37℃, 1 h; L: 37℃, 10 h.


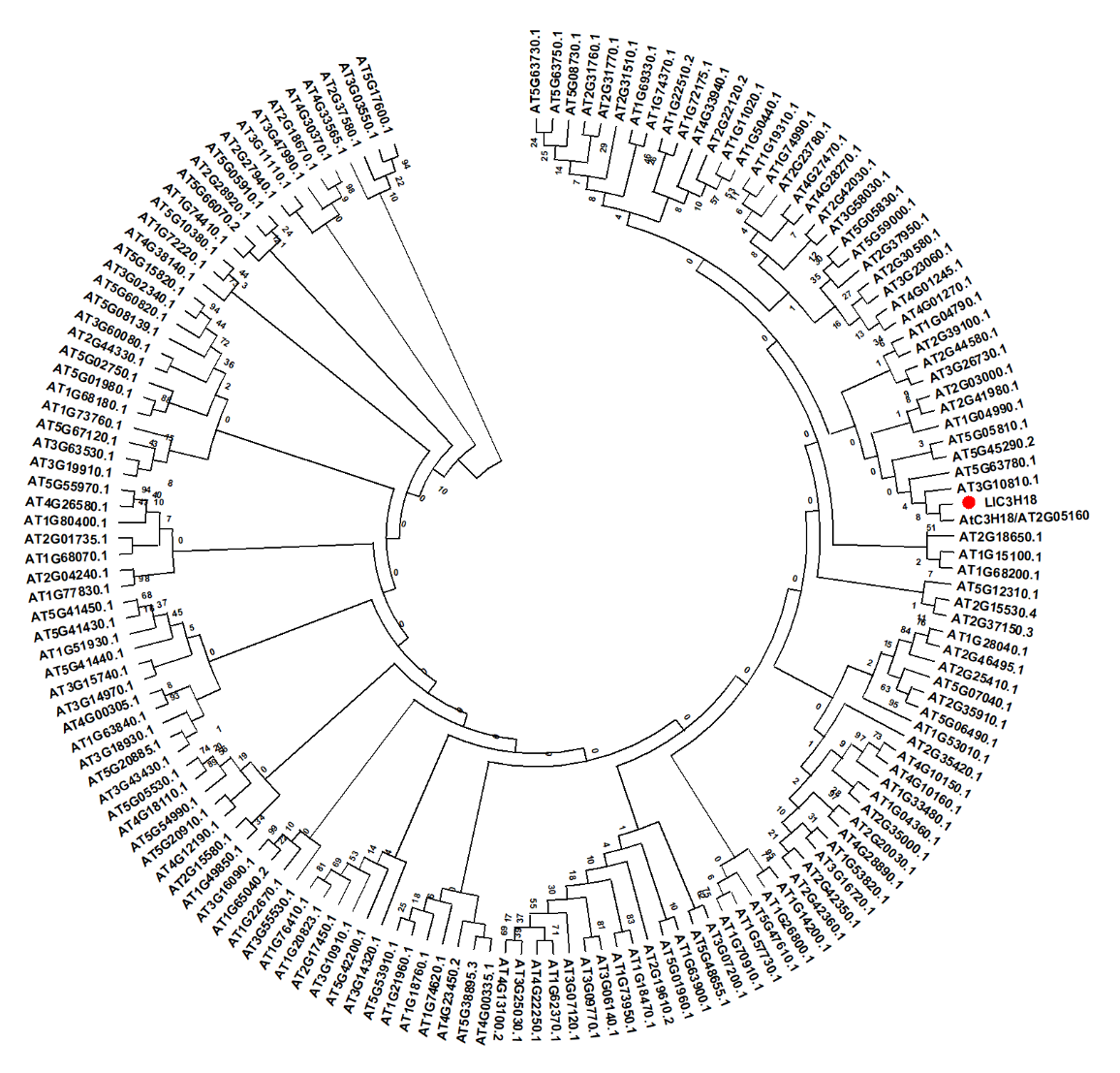


**Fig. S2** Phylogenetic analysis of LlC3H18 and CCCH-type proteins of Arabidopsis. The evolutionary tree was assembled in MEGA 7.0 with a neighbor-joining method (bootstrap replicates, *n* = 1,000).


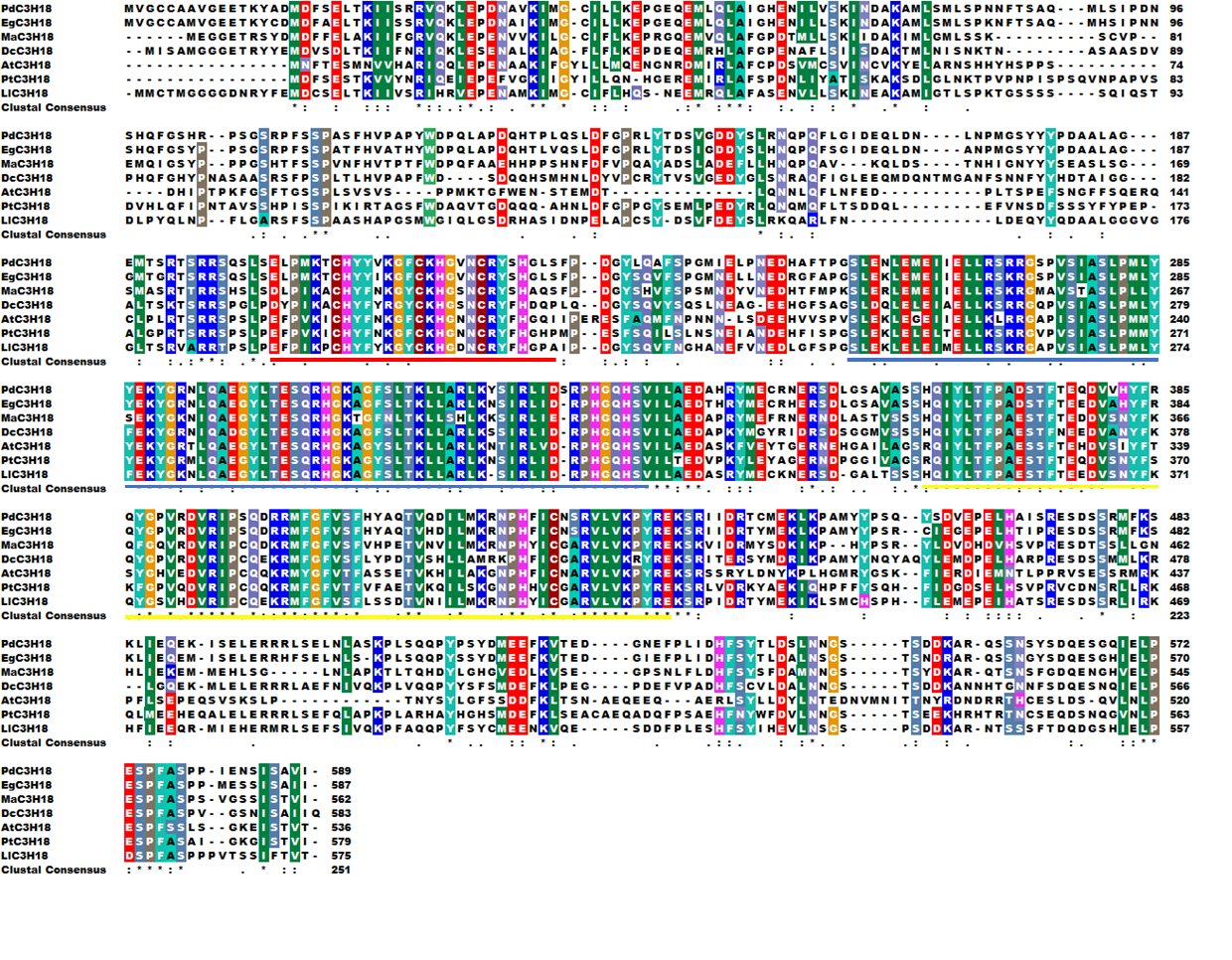


**Fig. S3** Sequence analysis of LlC3H18. The CCCH domain is labeled with red line, the LOTUS domain is labeled with blue line, and the RRM domain is labeled with yellow line. CCCH, CCCH zinc-finger motif; LOTUS, Limkain, Oskar, and TUdor-containing proteins 5 and 7; RRM, RNA-recognition motif. PdC3H18 (*Phoenix dactylifera*, XP_008800632.1); EgC3H18 (*Elaeis guineensis*, XP_010907182.1); DcC3H18 (*Dioscorea cayenensis*, XP_039146376.1); MaC3H18 (*Musa acuminata*, XP_009394377.1); AtC3H18 (*Arabidopsis thaliana*, AT2G05160); PtC3H18 (*Populus trichocarpa*, XP_024441083.1); PmC3H18 (*Prunus mume*, XP_008229903.1).


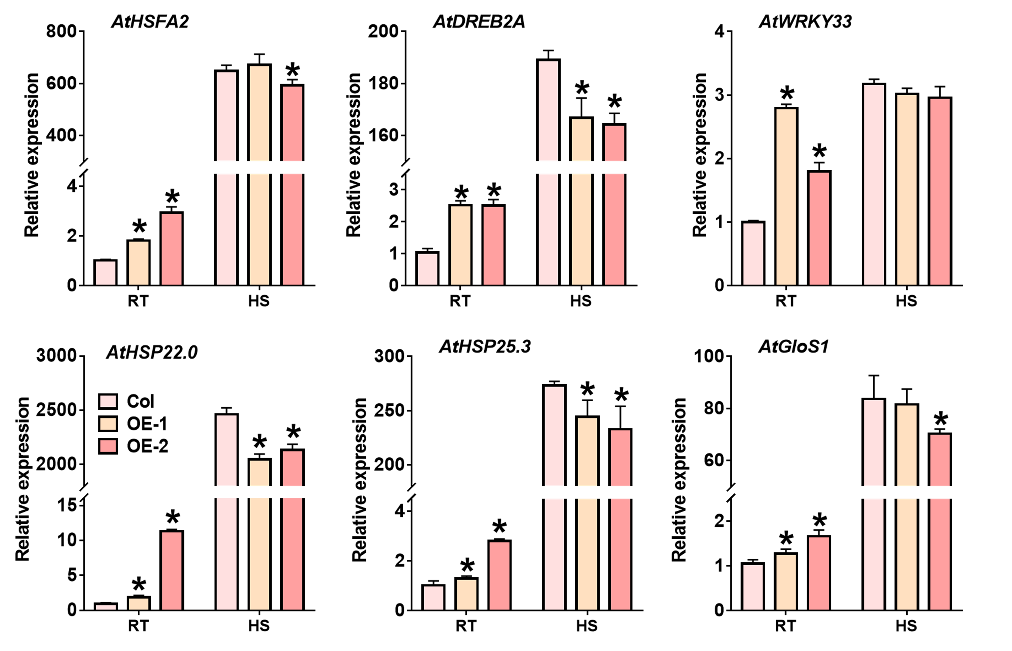


**Fig. S4** Expression analysis of heat-related genes in the wild-type and *LlC3H18* transgenic plants under normal and heat stress conditions. Data are the mean ± SD of three replicates (Student’s *t*-test, * P < 0.05).


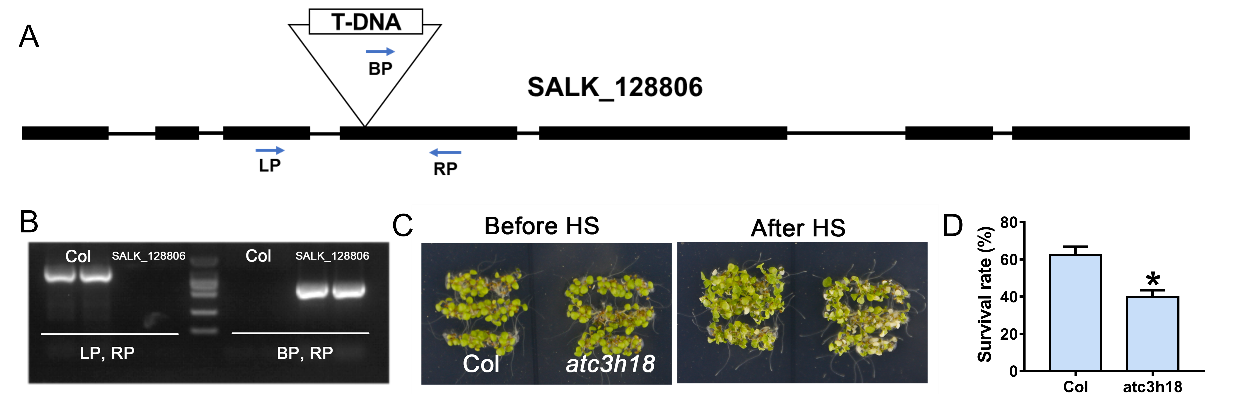


**Fig. S5** Identified T-DNA insertion *atc3h18* mutant and detection its thermotolerance. (A) Position of SALK_128806 T-DNA insertion in the *AtC3H18* gene and primers (FP, RP, and BP) used for PCR. (B) PCR analysis of the T-DNA insertion in wild-type and *atc3h18* mutant. (C) The 5-d-old seedlings were directly exposed to 45℃ conditions for 1 h to detect their thermotolerance ability; the figure is a photo image taken after 7 days of recovery at 22℃. (D) The survival rate, measured after 7 days of heat stress (HS). Bars are the mean ± SD of three independent experiments (Student’s *t*-test, *P < 0.05).


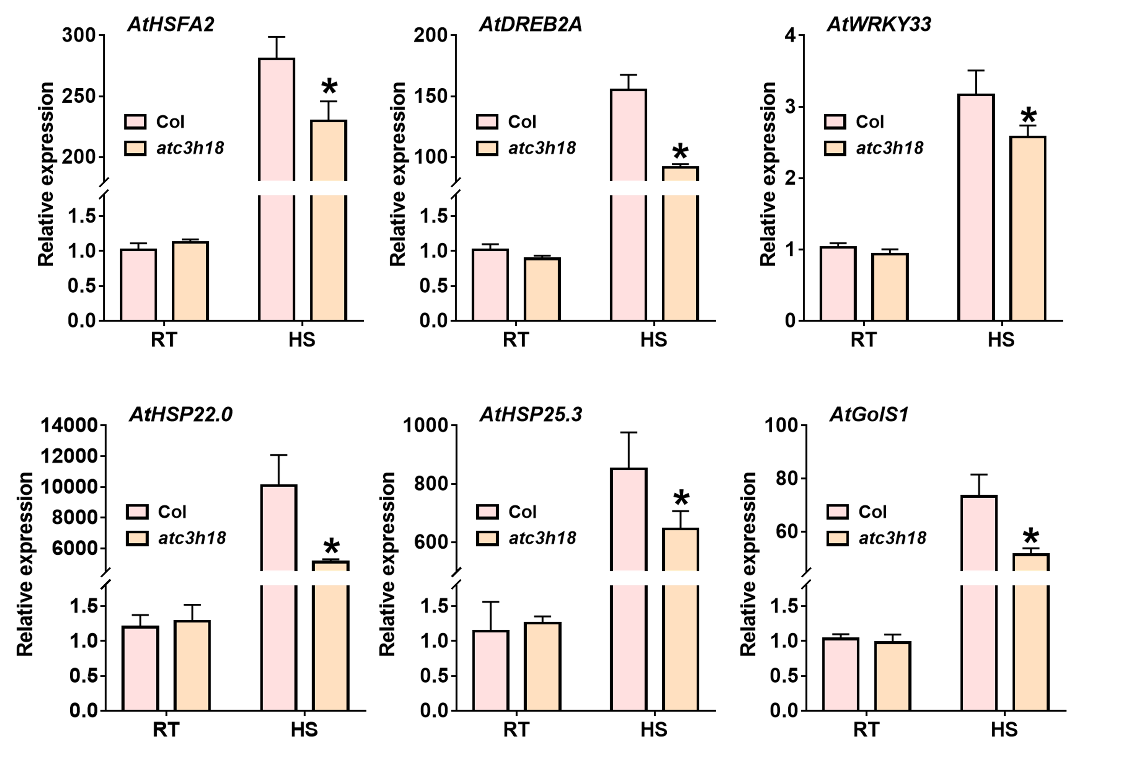


**Fig. S6** Expression analysis of heat-related genes in the wild-type and *atc3h18* mutant plants under normal and heat stress conditions. Data are the mean ± SD of three replicates (Student’s *t*-test, * P < 0.05).
